# ADAR1-mediated RNA editing of SCD1 drives drug resistance and self-renewal in gastric cancer

**DOI:** 10.1038/s41467-023-38581-8

**Published:** 2023-05-19

**Authors:** Tin-Lok Wong, Jia-Jian Loh, Shixun Lu, Helen H. N. Yan, Hoi Cheong Siu, Ren Xi, Dessy Chan, Max J. F. Kam, Lei Zhou, Man Tong, John A. Copland, Leilei Chen, Jing-Ping Yun, Suet Yi Leung, Stephanie Ma

**Affiliations:** 1 School of Biomedical Sciences, Li Ka Shing Faculty of Medicine, The University of Hong Kong, Hong Kong, China; 2 The University of Hong Kong – Shenzhen Hospital, Shenzhen, Guangdong China; 3 Department of Pathology, Sun Yat-Sen University Cancer Centre, Guangzhou, Guangdong China; 4 Department of Pathology, School of Clinical Medicine, Li Ka Shing Faculty of Medicine, The University of Hong Kong, Queen Mary Hospital, Hong Kong, China; 5 Cancer Science Institute of Singapore, National University of Singapore, Singapore, Singapore; 6 School of Biomedical Sciences, The Chinese University of Hong Kong, Hong Kong, China; 7 Department of Cancer Biology, Mayo Clinic Florida, Jacksonville, FL USA; 8 NUS Centre for Cancer Research, Yong Loo Lin School of Medicine, National University of Singapore, Singapore, Singapore; 9 The Jockey Club Centre for Clinical Innovation and Discovery, Li Ka Shing Faculty of Medicine, The University of Hong Kong, Hong Kong, China

**Keywords:** Gastric cancer, Cancer stem cells, Prognostic markers

## Abstract

Targetable drivers governing 5-fluorouracil and cisplatin (5FU + CDDP) resistance remain elusive due to the paucity of physiologically and therapeutically relevant models. Here, we establish 5FU + CDDP resistant intestinal subtype GC patient-derived organoid lines. JAK/STAT signaling and its downstream, adenosine deaminases acting on RNA 1 (ADAR1), are shown to be concomitantly upregulated in the resistant lines. ADAR1 confers chemoresistance and self-renewal in an RNA editing-dependent manner. WES coupled with RNA-seq identify enrichment of hyper-edited lipid metabolism genes in the resistant lines. Mechanistically, ADAR1-mediated A-to-I editing on 3’UTR of stearoyl-CoA desaturase (SCD1) increases binding of KH domain-containing, RNA-binding, signal transduction-associated 1 (KHDRBS1), thereby augmenting *SCD1* mRNA stability. Consequently, SCD1 facilitates lipid droplet formation to alleviate chemotherapy-induced ER stress and enhances self-renewal through increasing β-catenin expression. Pharmacological inhibition of SCD1 abrogates chemoresistance and tumor-initiating cell frequency. Clinically, high proteomic level of ADAR1 and SCD1, or high SCD1 editing/ADAR1 mRNA signature score predicts a worse prognosis. Together, we unveil a potential target to circumvent chemoresistance.

## Abstract (web summary)

Resistance to 5-fluorouracil and cisplatin (5FU + CDDP) presents a major issue in patients with gastric cancer. Here, the authors establish 5FU + CDDP resistant intestinal gastric cancer patient-derived organoids and identify JAK/STAT-ADAR1 altered lipid metabolism as a regulator of chemoresistance.

## Introduction

Gastric cancer (GC) is a therapeutically recalcitrant disease, accounting for more than 750,000 deaths in 2020^1^. To combat GC, 5-fluorouracil (5FU)- and platinum-based combination chemotherapy are often administered in addition to surgical resection^2^. Indeed, the combination of 5FU and cisplatin (CDDP) along with other chemotherapy applied in perioperative setting can improve survival compared to surgery alone^3–5^. However, the emergence of acquired chemoresistance eventually curtails long-term clinical benefits. The myriad mechanisms driving chemoresistance, compounded with GC constituting of various subtypes obscure the identification of targets to override chemoresistance. Furthermore, the majority of experiments investigating chemoresistance were conducted with single chemotherapy, contradictory to the combination chemotherapy commonly applied in the clinical setting. Various histological and molecular classification have been established; however, the subtype-specific vulnerability has been relatively unexploited for clinical advances. Accordingly, there is an unmet need to discern subtype-specific novel targets to circumvent combination chemoresistance.

RNA editing, a post-transcriptional RNA modification, refers to the enzymatic conversation of RNA nucleotide. RNA editing enriches epitranscriptomic diversity by governing processes such as RNA splicing, protein recoding, miRNA binding and biogenesis, mRNA stability and mRNA localization^6^. Adenosine-to-inosine (A-to-I) editing, the most prevalent form of RNA editing in human, has an emerging role in cancer progression^7,8^. It has also been linked to activate cancer stem cells^9^ and protect against the activation of innate immunity^10^. Among the ADAR family members, ADAR1 and ADAR2 have been shown to promote and attenuate GC progression, respectively, in an RNA editing-dependent manner^11^. More recently, an RNA editing signature has been demonstrated to guide GC chemotherapy in the clinic^12^. Yet, whether ADAR1 mediates chemoresistance in GC remains unknown and the effects of specific altered editing event(s) that may functionally contribute to GC chemoresistance have not been identified. Pivotal to chemoresistance is the self-renewal ability of cancer cells that can fuel the repopulation of tumor after treatment^13^. Studies have demonstrated that ADAR1 contributes to the cancer stemness properties in blood cancer^14,15^ and brain cancer^16^ but not in GC. Together, the potential clinical implication of RNA editing necessitates the need to explore its role in chemoresistance and self-renewal.

Cancer cell lines have been the core of mechanistic studies to understand chemoresistance. However, the extended period of culture in vitro coupled with inadequate information regarding the tumor they were derived from challenge how well these cell lines resemble the tumor physiological condition. As such, using GC patient-derived organoid cultures that faithfully mirror the histological and molecular features, along with the drug response of the patients they were derived from^17^, may illuminate unreported mechanistic insights modulating chemoresistance.

In this work, we train GC patient-derived organoids of the intestinal subtype to be resistant to 5FU + CDDP treatment. Transcriptome profiling (RNA-seq) reveals enrichment of interferon and JAK/STAT signaling in the resistant organoid lines. Functional assays depict that adenosine deaminases acting on RNA 1 (ADAR1), a target of JAK/STAT, endows chemoresistance in an RNA editing-dependent manner. Whole-exome sequencing (WES) coupled with RNA-seq unmask an aberrant A-to-I RNA editome in the resistant organoid lines. Stearoyl-CoA desaturase-1 (SCD1), a target of ADAR1 and a critical player in lipid metabolism, confers chemoresistance and self-renewal while pharmacological inhibition of SCD1 abrogates its oncogenic influence, efficiently blocking GC self-renewal and stemness as well as reverting chemoresistance. Together, our study discovers a targetable mechanism driving GC chemoresistance.

## Results

### 5FU + CDDP drug resistant gastric organoids exhibit interferon/JAK/STAT signaling activation leading to induction of ADAR1 expression

**Fig. 1:**
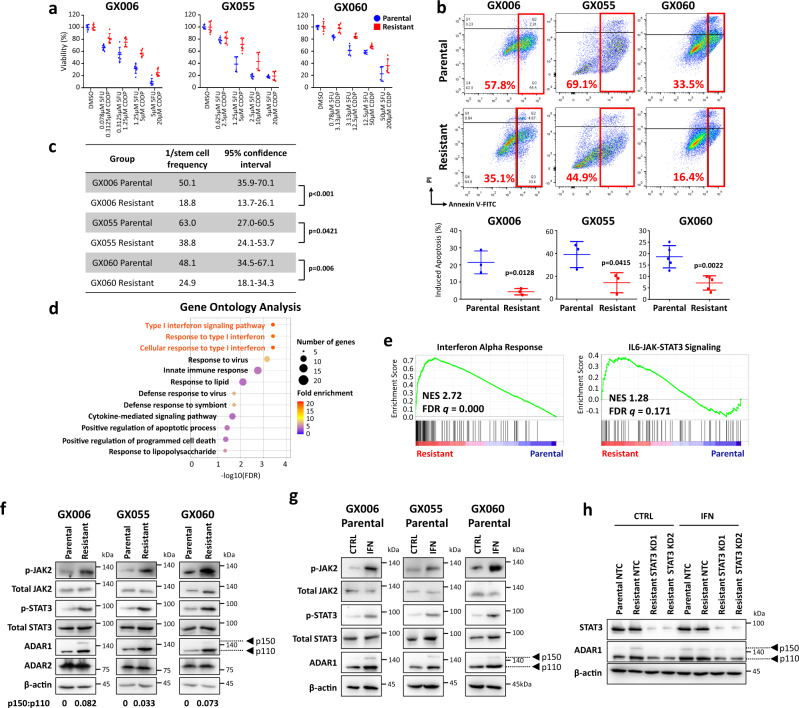
5FU + CDDP drug resistant gastric organoids exhibit interferon/JAK/STAT signaling activation leading to induction of ADAR1 expression. GC patient-derived organoids of intestinal subtype GX006, GX055 and GX060 were trained with increasing concentrations of 5-fluorouracil (5FU) and cisplatin (CDDP) combination to develop 5FU + CDDP resistant lines. **a** CellTiter-Glo analysis showing the viability of parental versus 5FU + CDDP resistant organoids following treatment in various concentrations of 5FU + CDDP combinations. **b** Annexin V-PI analysis showing the percentage of apoptotic cells in parental versus 5FU + CDDP resistant organoids following treatment in 1.25 µM 5FU + 5 µM CDDP (GX006) or 5 µM 5FU + 20 µM CDDP (GX055 and GX060). **c** In vitro limiting dilution spheroid formation and tumor-initiating cell frequency calculation in parental versus 5FU + CDDP resistant organoids. **d, e** Gene Ontology (GO) (**d**) and Gene Set Enrichment Analysis (GSEA) (**e**) of differentially expressed genes identified by RNA-seq data found enrichment of interferon signaling and its downstream JAK/STAT signaling in the 5FU + CDDP resistant organoids as compared to parental controls. **f** Western blot for phosphorylated and total JAK2, phosphorylated and total STAT3, ADAR1 and ADAR2 in the three paired parental and 5FU + CDDP resistant organoid lines. β-actin served as a loading control. **g** Western blot for phosphorylated and total JAK2, phosphorylated and total STAT3 and ADAR1 in the three parental organoid lines with or without interferon (1000 U/mL) treatment for 24 hours. **h** Western blot for total STAT3 and ADAR1 in the GX006 parental and GX006 5FU + CDDP resistant organoid lines stably transduced with non-target control (NTC) or STAT3 shRNA knockdown (clones 1 and 2) after treatment with vehicle control (CTRL) or 1000 U/mL interferon (IFN) for 24 hours. β-actin served as a loading control. Images representative of *n* = 3 independent experiments. (**a**) *n* = 2 independent experiments; (**b**) *n* = 3 independent experiments for GX006, GX005 and *n* = 5 independent experiments for GX060; (**c, f, g, h**) *n* = 3 independent experiments. Significance were calculated by (**b**) unpaired two-tailed student t-test; (**c**) one-sided extreme limiting dilution analysis. Data was presented as mean ± standard deviation. NES for normalized enrichment score, FDR for false discover rate. Source data are provided as a Source Data file.

Intestinal GC is the most common subtype of GC, accounting for over 50% of all GC patients. Analysis of TCGA-STAD data using Lauren’s subtype, a classification commonly applied in the clinic^18^, revealed that chemotherapy is beneficial for patients with intestinal subtypes, yet those patients that developed acquired chemoresistance had worse prognosis (Supplementary Fig. 1), indicating that reversing chemoresistance may yield potential therapeutic benefits to patients with intestinal subtype. To understand intestinal subtype-specific mechanisms driving chemoresistance, GC patient-derived organoids of intestinal subtype GX006, GX055, and GX060 were trained with continuous increasing concentrations of 5-fluorouracil (5FU) and cisplatin (CDDP) combination, beginning from IC_10_ to IC_50_, to develop 5FU + CDDP resistant lines. The same volume of DMSO was added to parental organoids as mock controls. Acquired 5FU + CDDP chemoresistant properties were functionally demonstrated by increased proliferation (Fig. 1a), reduced apoptosis (Fig. 1b, Supplementary Fig. 2) and enhanced tumor-initiating capacity (Fig. 1c) in the resistant compared to parental controls. Whole-exome sequencing (WES) revealed no significant alterations at the genomic level (Supplementary Fig. 3), while pathway enrichment analysis on differentially expressed genes identified by RNA-seq uncovered significant enrichment of interferon signaling (e.g., type I interferon signaling pathway, response to type I interferon, cellular response to type I interferon, interferon alpha response) and insignificant but positive enrichment of its downstream JAK/STAT signaling in the resistant organoid lines (Fig. 1d-e). This observation was subsequently confirmed by western blot where p-JAK2 and p-STAT3 were both found to be enhanced in the 5FU + CDDP resistant organoid lines as compared to parental controls (Fig. 1f). ADAR1 has been shown to be regulated by interferon through the JAK/STAT pathway in glioblastoma and leukemic stem cells^15,16^. Western blot analysis for ADAR1 and ADAR2, the two RNA-editing enzymes known to be expressed in gastric cancer^11^, found the resistant organoid lines to express enhanced ADAR1 (Fig. 1f). Note ADAR3 is expressed specifically in the brain and has no documented deaminase activity in gastric cancer and thus was not studied. To further explore if ADAR1 functions as a downstream effector of interferon/JAK/STAT in gastric cancer, we treated organoid lines with type I interferon. Unlike the previous report that interferon induces the expression of p150 ADAR1 but not p110 ADAR1, we observed increase of both p150 and p110 ADAR1 following the addition of interferon to the organoid lines (Fig. 1g, Supplementary Fig. 4). Suppression of STAT3 signaling by shRNA or inhibitor (BBI608) in 5FU + CDDP resistance organoid lines reduced expression of ADAR1 (Supplementary Fig. 5a-b), even in the presence of interferon activation (Fig. 1h). These collectively suggest that ADAR1 is upregulated in 5FU + CDDP resistance organoid lines through activation of interferon/JAK2/STAT3 signaling.

### ADAR1 enzyme promotes drug resistance and self-renewal in 5FU + CDDP-resistant organoids

**Fig. 2:**
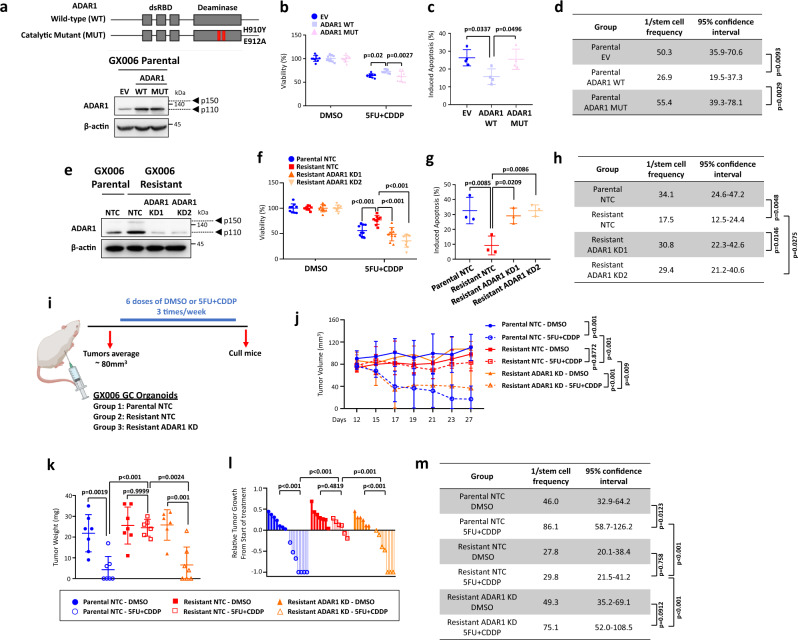
ADAR1 promotes chemoresistance and self-renewal in 5FU + CDDP-resistant organoids. **a** Schematic diagram of ADAR1 wild-type (WT) and catalytically-dead mutant (MUT) with point mutations in the deaminase domain illustrated. Western blot for ADAR1 in GX006 parental organoid lines stably transduced with empty vector (EV) control, ADAR1 WT or ADAR1 MUT. β-actin served as a loading control. **b–d** CellTiter-Glo analysis (**b**) and Annexin V-PI analysis (**c**) in the absence or presence of 1.25 µM 5FU + 5 µM CDDP, and in vitro limiting dilution spheroid formation and tumor-initiating cell frequency calculation (**d**) in GX006 parental organoid lines stably transduced with EV, ADAR1 WT or ADAR1 MUT. **e** Western blot for ADAR1 in GX006 parental and GX006 5FU + CDDP resistant organoid lines stably transduced with non-target control (NTC) or ADAR1 shRNA knockdown (clones 1 and 2). β-actin served as a loading control. **f–h** CellTiter-Glo analysis (**f**) and Annexin V-PI analysis (**g**) in the absence or presence of 5FU + CDDP, and in vitro limiting dilution spheroid formation and tumor-initiating cell frequency calculation (**h**) in GX006 parental and GX006 5FU + CDDP resistant organoid lines stably transduced with NTC or ADAR1 shRNA (clones 1 and 2). **i** Schematic diagram of treatment regimen comparing GX006 parental and resistant organoid lines stably transduced with NTC or ADAR1 shRNA (clones 1) injected into NSG mice subcutaneously. **j, k** Volume (**j**) and weight (**k**) of tumors derived from the indicated cell lines at end point. **l** Waterfall plot showing the response of each tumor in each group at end point. **m** Ex vivo limiting dilution assay of tumors harvested from each group to evaluate tumor-initiating cell frequency. (**a,** **b,** **d,** **e,** **f,** **g,** **h**) n = 3 independent experiments; (**c**) *n* = 4 independent experiments); (**i–m**), n = 6-7 mice. Significance were calculated by (**b, f, j)** two-way ANOVA; (**c, g, k, l**) by one-way ANOVA; (**d,** **h,** **m**) by one-sided extreme limiting dilution analysis. Data was presented as mean ± standard deviation. EV for empty vector control, WT for wild-type, MUT for catalytically-dead mutant, NTC for non-target control, ADAR1 KD1 and KD2 for shRNA knockdown (clones 1 and 2). ns for not significant. Source data are provided as a Source Data file. Illustration for (**i**) was created using BioRender.com.

ADAR1 exists in two isoforms, p110 and p150, which predominantly locate in the nucleus and cytoplasm, respectively. Since we observed a much higher expression of p110 ADAR1 in our gastric cancer organoid lines (ratio of p150 ADAR1 to p110 ADAR1 < 0.1, Fig. 1f), we decided to first focus on the function of p110 ADAR1 isoform in governing resistant to 5FU + CDDP. To understand if ADAR1 enzymatic activity is responsible for changes in GC 5FU + CDDP resistance, we transduced a patient-derived GC organoid (GX006) with an empty vector (EV) control, a wild-type (WT) ADAR1 (p110 isoform), or an catalytically-dead mutant (MUT) ADAR1 (p110 isoform) that contains point mutations in its catalytic site (H910Y and E912A) (Fig. 2a)^11^. GC organoids transduced with WT ADAR1 displayed increased proliferation, decreased apoptosis and enhanced self-renewal relative to EV control. In contrast, catalytically-dead ADAR1 reduced proliferation, induced apoptosis and reduced sphere formation relative to WT ADAR1, exhibiting a similar functional capacity as EV control, suggesting the catalytically-dead ADAR1 functions as a dominant negative (Fig. 2b-d, Supplementary Fig. 6a). In an equivalent manner, we also suppressed ADAR1 by lentiviral-based shRNA knockdown in 5FU + CDDP GC resistant organoids (GX006) where the same phenomenon was consistently observed (Fig. 2e-h, Supplementary Fig. 6b). We also interrogated ADAR1 dependency in driving 5FU + CDDP chemoresistance in tumor xenograft experiments. GC parental and resistant organoids transduced with a control shRNA encoding a non-targeting sequence or GC resistant organoids transduced with shADAR1 were transplanted subcutaneously into immunocompromised mice. When tumors reached an average of 80mm^3^, mice were randomized and treated with either DMSO or 5FU + CDDP (Fig. 2i). As expected, resistant organoid xenografts displayed resistance to 5FU + CDDP treatment as compared to parental organoid xenografts; while knockdown of ADAR1 in the resistant organoid would revert this ability. A notable change in tumor volume, tumor weight, relative tumor growth and tumor-initiating capacity was noted (Fig. 2j-m). Continuous knockdown of ADAR1 in the xenografted tumors was further confirmed by immunohistochemistry (Supplementary Fig. 6c). Similar functional findings were also consistently noted when ADAR1 was overexpressed in NUGC3 gastric cancer cell, when ADAR1 was repressed in MKN28 gastric cancer cell, or when STAT3 was repressed in GC 5FU + CDDP resistant organoid (Supplementary Figs. 5c-k and 7). To determine the ADAR1 isoform contributing to the resistance to 5FU + CDDP, we also overexpressed p150 ADAR1 in the parental organoid and compared it to the effect of p110 ADAR1 (Supplementary Fig. 8a). Overexpression of p150 ADAR1 showed a slight improvement to 5FU + CDDP treatment compared to EV control, though the effect was significantly lower compared to p110 ADAR1 (Supplementary Fig. 8b-d). The expression of p150 ADAR1 after overexpression remained lower than that of the endogenous p110 ADAR1 expression, suggesting that p110 ADAR1 is the predominant driving factor in the GC 5FU + CDDP-resistant organoids. Therefore, we focused on p110 ADAR1 in understanding the mechanism of resistance to 5FU + CDDP.

### ADAR1-mediated RNA editing of *SCD1* promotes its expression

**Fig. 3:**
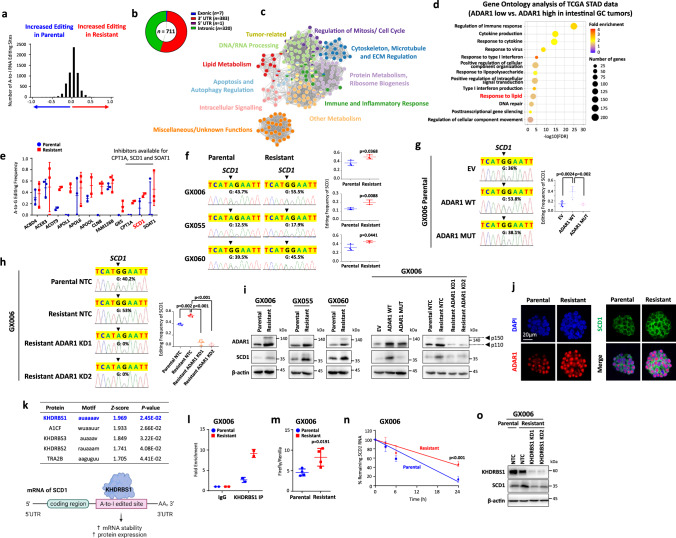
ADAR1-mediated RNA editing of SCD1 promotes its expression. **a** Distribution of putative A-to-I RNA editing sites (n = 6025). **b-c** Distribution of A-to-I RNA editing events hyper-edited in 5FU + CDDP resistant organoid lines as categorized by regions of the RNA transcript (**b**) and biological processes (**c**). **d** Gene ontology analysis of differentially regulated genes by comparing gastric cancer patients of intestinal subtype (TCGA-STAD) with high *ADAR1* or low *ADAR1* expression (stratified by median *ADAR1* expression). **e** Putative hyper-edited genes involved in lipid metabolism from GX006, GX055 and GX060. *n* = 3 biologically independent samples. **f-h** Sequence chromatograms of the *SCD1* transcript in the indicated cell groups. Dot plots represent editing levels of *SCD1*. **i** Western blot for ADAR1 and SCD1 expression in the indicated cell groups. β-actin served as a loading control. **j** Immunofluorescence images showing concomitant high expression of ADAR1 with SCD1. Scale bar, 20 µm. **k** Top 5 RNA binding proteins predicted to bind to *SCD1* 3’UTR A-to-I editing sites by RBPmap. Illustration of binding of KHDRBS1 on to *SCD1* 3’UTR and the potential effect of A-to-I editing on the binding sites on *SCD1* RNA. **l** immunoprecipitation binding assay of KHDRBS1 in parental or resistant organoids (GX006). **m** Luciferase reporter assay with *SCD1* 3’UTR in parental or resistant organoids (GX006). **n** Stability of *SCD1* RNA following Actinomycin D treatment (10 µg/mL) for 3, 6, or 24 hours. Lines were linear regression of the data. **o** Western blot for KHDRBS1 and SCD1 expression in GX006 parental and GX006 5FU + CDDP resistant organoid lines stably transfected with NTC or KHDRBS1 shRNA (clones 1 and 2). (**f, h, i, j, o**) *n* = 3 independent experiments; (**g, m, n**) *n* = 4 independent experiments; (**l**) *n* = 2 independent experiments. Significance were calculated by (**e, f, l m**) unpaired two-tailed student t-test; (**g**–**h**) one-way ANOVA; (**n**) two-way ANOVA. Data was presented as mean ± standard deviation. EV for empty vector control, WT for wild-type, MUT for catalytically-dead mutant, NTC for non-target control, ADAR1 KD1 and KD2 for shRNA knockdown (clones 1 and 2), KHDRBS1 KD1 and KD2 for shRNA knockdown (clone 1 and 2). Source data are provided as a Source Data file. Illustration for (**k**) was created using BioRender.com.

To elucidate ADAR1-mediated A-to-I RNA editing in driving GC 5FU + CDDP chemoresistance, we interrogated the RNA editomes of all three paired parental and resistant GC organoids by RNA-seq (Supplementary Fig. 9a). To eliminate false positives resulting from potential DNA contamination, we matched whole-exome sequencing data from the same samples. We examined the distribution of variants found exclusively on RNA and found that the most common variants were A-to-G (A-to-I) and C-to-T (C-to-U), which is consistent with our previous report showing that A-to-I RNA editing is the most prevalent type of RNA editing events in gastric cancer^11^ (Supplementary Fig. 9b). Next, we examined the A-to-I editing landscape and discovered there is an enrichment of A-to-I changes in the resistant organoids as compared to parental organoids, suggesting the augmented RNA editing may drive chemoresistance (Fig. 3a), with 5FU + CDDP resistant organoids-enriched editing events most enriched in the 3’UTR regions as compared to other exonic and intronic regions (Fig. 3b, Supplementary Data 1). Analysing the biological process of genes with higher editing levels in resistant as compared to parental organoids showed involvement in various processes including lipid metabolism (Fig. 3c). Surprisingly, when grouping gastric cancer patients from the TCGA cohort (TCGA-STAD) based on *ADAR1* expression, we also observed enrichment of lipid metabolism in addition to the immune-related response in samples with high *ADAR1* expression (Fig. 3d). Similarly, pathway enrichment analysis on differentially expressed genes identified enrichment of lipid metabolism in resistant organoids compared to parental controls (Fig. 1d). These suggested that lipid metabolism may be regulated in part by A-to-I editing through ADAR1. Of the 12 putative hyper-edited genes involved in lipid metabolism, only carnitine palmitoyltransferase 1 A (CPT1A), SCD1 and sterol O-Acyltransferase 1 (SOAT1) are targetable by commercially available inhibitors (Fig. 3e). CPT1A inhibitor, Etomoxir, has been shown to demonstrate hepatotoxicity in clinical trial^19^ and thus was not considered for further study here. Between SCD1 and SOAT1, we found that *SCD1* expression level is positively correlated with a cancer stemness signature^20^ and poor prognosis in GC patients treated with chemotherapy, thereby suggesting SCD1 to be a more interesting and likely downstream effector of ADAR1 to promote cancer stemness and chemoresistance (Supplementary Fig. 10). SCD1 is one of the key rate-limiting metabolism enzymes regulating the synthesis of unsaturated fatty acids and lipid. Therefore, we investigated the change in fatty acids composition and lipid metabolism in resistant organoids. Through lipidomics analysis, we observed an overall increased level of unsaturated fatty acids and key substrate for de novo lipid synthesis, glycerol-3-phosphate, in all three GC-resistant organoids (Supplementary Fig. 11a-b). The metabolism of lipid also shifted to anabolism as indicated by decreased palmitate utilization in resistant organoids (Supplementary Fig. 11c). This data collectively suggested a change from utilization to storage and synthesis of lipid in resistant organoids. We identified one prevalent 5FU + CDDP resistant GC organoid-specific A-to-I edits in the 3’UTR of *SCD1* (chromosome 10:102,121,601) and confirmed an increase in editing frequency at this site in RNA while editing being absent in DNA by Sanger sequencing (Fig. 3f, Supplementary Fig. 12). When GC parental organoids (GX006) were transduced with WT ADAR1, RNA editing events were enhanced, but not when EV control or MUT ADAR1 was transduced (Fig. 3g). Consistently, RNA editing events were diminished in resistant organoids upon transduction with two independent shADAR1 sequences, but not non-targeting control shRNA (Fig. 3h). To demonstrate ADAR1-mediated RNA editing of *SCD1* is responsible for enhanced SCD1 expression, we performed rescue experiments whereby WT and MUT ADAR1 were overexpressed in parental organoids or shADAR1 knockdown was transduced in resistant organoids. While both WT and MUT ADAR1 were expressed at equivalent levels, only WT ADAR1 rescued SCD1 expression in parental GC organoids. Targeting ADAR1 expression in 5FU + CDDP resistant GC organoids reduced the protein expression of both ADAR1 and SCD1, suggesting ADAR1 as an upstream regulator of SCD1 (Fig. 3i). By dual-color immunofluorescence, we were also able to see the heightened expression of ADAR1 and SCD1 in the resistant versus parental organoids. The strong localization of ADAR1 to the nucleus further confirmed the predominant isoform is ADAR1 p110 (Fig. 3j). Next, we explored on the consequence of *SCD1* editing and how that contributes to its increased protein expression. Based on analysis of potential RNA proteins binding to *SCD1* 3’UTR using RBPmap^21^, RNA binding protein KHDRBS1 exhibited the highest score to bind to the 3’UTR specific to the A-to-I RNA edited site of *SCD1* (Fig. 3k). In situ prediction of *SCD1* 3’UTR secondary structure using RNAfold^22^ showed double strand around the site of A-to-I editing, confirming the potential of ADAR1 and KHDRBS1 binding (Supplementary Fig. 13a). Immunofluorescence staining and subfractionation of GC organoids showed that KHDRBS1 localized to the nucleus, suggesting the location of A-to-I editing by ADAR1 and binding of KHDRBS1 occurred both in the nucleus (Supplementary Fig. 13b-c). The binding of KHDRBS1 to A-to-I editing site of 3’UTR of *SCD1* RNA was confirmed through RNA immunoprecipitation (Fig. 3l, Supplementary Fig. 13d). Using luciferase reporter and RNA stability assays, the stability of *SCD1* RNA were increased following A-to-I editing (Fig. 3m-n, Supplementary Fig. 13e-g). Knockdown of KHDRBS1 in resistant organoids decreased SCD1 protein level and SCD1 3’UTR luciferase signal, supporting the role of KHDRBS1 in the regulation of *SCD1* RNA stability leading to an increase in protein level (Fig. 3o, Supplementary Fig. 13h). To exclude the possibility that A-to-I editing increased SCD1 protein expression via increasing transcription, we measured *SCD1* mRNA expression in resistant organoids, following overexpression of ADAR1 or knockdown of ADAR1. We observed no significant difference in all conditions (Supplementary Fig. 13i-k). Again, consistent findings could also be seen in NUGC3 and MKN28 cells, further lending evidence to show that ADAR1 regulated the expression of SCD1 through A-to-I editing on 3’UTR of *SCD1* (Supplementary Fig. 14).

### ADAR1-mediated upregulation of SCD1 drives chemoresistance in gastric cancer

**Fig. 4:**
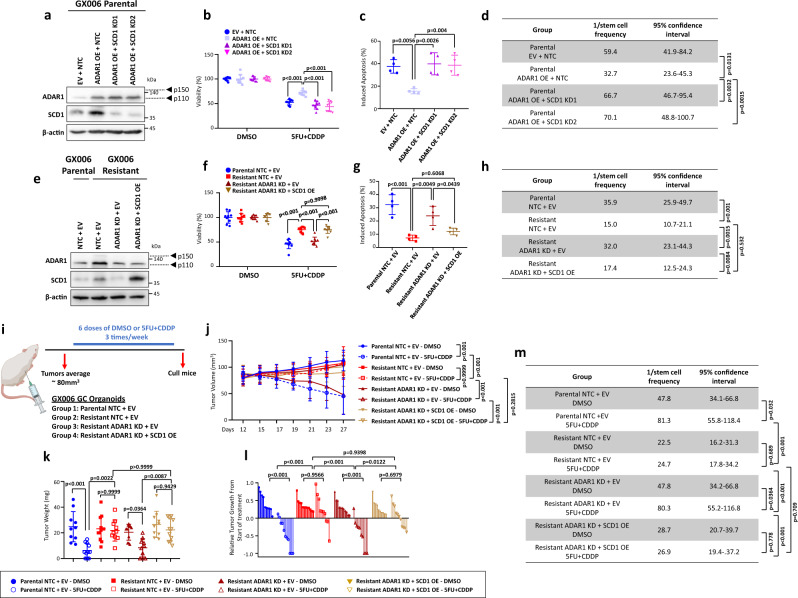
ADAR1-mediated upregulation of SCD1 drives chemoresistance in gastric cancer. **a** Western blot for ADAR1 and SCD1 expression in GX006 parental organoids with or without ADAR1 overexpressed and with or without SCD1 concomitantly repressed. **b–d** CellTiter-Glo analysis of cell viability (**b**), Annexin V-PI analysis of apoptotic cells (**c**) and in vitro limiting dilution spheroid formation and tumor-initiating cell frequency calculation (**d**) in GX006 parental organoid lines with or without ADAR1 overexpressed and with or without SCD1 concomitantly repressed. **e** Western blot for ADAR1 and SCD1 expression in GX006 parental and GX006 5FU + CDDP resistant organoid lines with or without ADAR1 repressed and with or without SCD1 concomitantly overexpressed. Images representative of *n* = 3 independent experiments. **f–h** CellTiter-Glo analysis of cell viability (**f**), Annexin V-PI analysis of apoptotic cells (**g**) and in vitro limiting dilution spheroid formation and tumor-initiating cell frequency calculation (**h**) GX006 parental and GX006 5FU + CDDP resistant organoid lines with or without ADAR1 repressed and with or without SCD1 concomitantly overexpressed. **i** Schematic diagram of treatment regimen comparing GX006 parental and GX006 5FU + CDDP resistant organoid lines with or without ADAR1 repressed and with or without SCD1 concomitantly overexpressed injected into NSG mice subcutaneously. **j, k** Volume (**j**) and weight (**k**) of tumors derived from the indicated cell lines at end point. **l** Waterfall plot showing the response of each tumor in each group at end point. **m** Ex vivo limiting dilution assay of tumors harvested from each group to evaluate tumor-initiating cell frequency. (**a–h**) *n* = 3 independent experiments; (**i–m**) *n* = 10–12 mice. Significance were calculated by (**b, f, j**) two-way ANOVA; (**c, g, k, l**) by one-way ANOVA; (**d, h, m**) by one-sided extreme limiting dilution analysis. Data was presented as mean ± standard deviation. EV for empty vector control, NTC for non-target control, OE for overexpression, SCD1 KD1 and KD2 for SCD1 shRNA knockdown (clones 1 and 2), ADAR1 KD for ADAR1 shRNA knockdown (clone 1). ns for not significant. Source data are provided as a Source Data file. Illustration for (**i**) was created using BioRender.com.

To determine if the loss-of-function of SCD1 phenocopied the loss of ADAR1, we used two independent shRNAs to knockdown SCD1 in ADAR1 overexpressing GC parental organoids and assessed their functional impact (Fig. 4a). Silencing of SCD1 in ADAR1 overexpressed GC parental organoids reduced proliferation, induced apoptosis and decreased spheroid formation in limiting dilution assays compared to non-targeting control shRNA, returning functional capacity levels back to parental organoids that were transduced with just controls (Fig. 4b-d, Supplementary Fig. 15a-b). On the other hand, resistant organoids that had ADAR1 stably repressed and SCD1 concomitantly overexpressed would exhibit increased proliferation, reduced apoptosis and enhanced spheroid formation ability, similar to the levels of those exhibited by resistant organoids transfected with controls (Fig. 4e-h). We also extended into in vivo animal studies whereby GC parental and resistant organoids transduced with controls or GC resistant organoids concomitantly transduced with shADAR1 and empty vector control or SCD1 overexpression were transplanted subcutaneously into immunocompromised mice. When tumors reached an average of 80mm^3^, mice were treated with either DMSO or 5FU + CDDP (Fig. 4i). While knockdown of ADAR1 in the resistant organoid would reduce tumor growth and tumor-initiating potential, SCD1 overexpression would reverse this phenotype (Fig. 4j-m). Immunohistochemical staining on serial sections further confirmed co-localization of ADAR1 and SCD1 in the resistant organoids, while concomitantly validating ADAR1 suppression and SCD1 overexpression in the xenografted tumors (Supplementary Fig. 15c). Similar functional findings were also consistently observed when ADAR1 was overexpressed in NUGC3 or when ADAR1 was repressed in MKN28 gastric cancer cells (Supplementary Fig. 16).

### SCD1 inhibitor sensitizes 5FU + CDDP-drug resistant gastric cancer to chemo-treatment and reduces tumor-initiating cell frequency

**Fig. 5:**
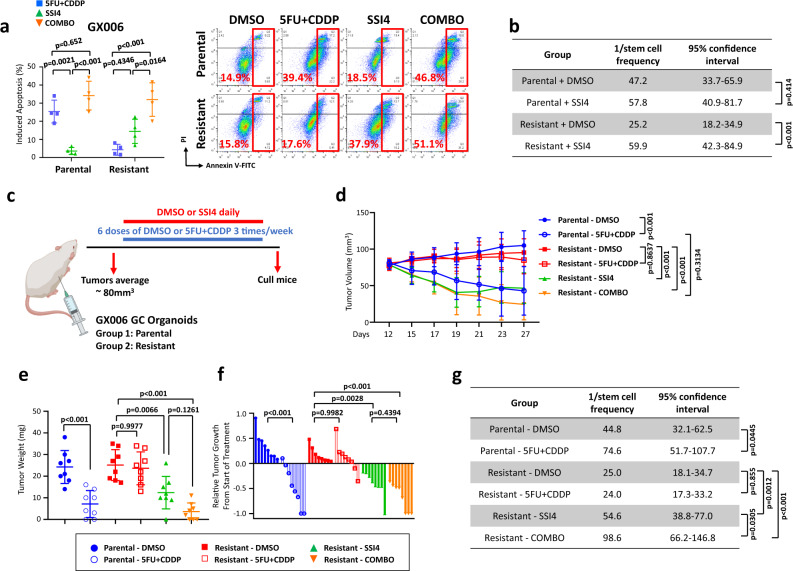
SCD1 inhibitor sensitizes 5FU + CDDP-drug resistant gastric cancer to chemo-treatment and reduces tumor-initiating cells frequency. **a, b** Functional assays investigating the effect of pharmacological inhibition of SCD1 using a SCD1 specific inhibitor SSI4 in GX006 parental and 5FU + CDDP resistant organoid lines. Comparisons include DMSO versus 5FU + CDDP versus SSI4 versus combination of 5FU + CDDP and SSI4 (COMBO) using Annexin V-PI apoptosis (**a**) and in vitro limiting dilution spheroid formation assays (**b**). **c** Schematic diagram of treatment regimen comparing GX006 parental and 5FU + CDDP resistant organoid lines treated with DMSO or 5FU + CDDP and in combination with vehicle or SSI4. **d, e** Volume (**d**) and weight (**e**) of tumors derived from the indicated treatment groups at end point. **f** Waterfall plot showing the response of each tumor in each treatment group at end point. **g** Ex vivo limiting dilution assay of HCC tumors harvested from each treatment group to evaluate tumor-initiating cell frequency. (**a**) *n* = 4 independent experiments; (**b**) *n* = 3 independent experiments; (**c–e**) *n* = 8 mice. Significance were calculated by (**a, d**) two-way ANOVA; (**b, g**) one-sided extreme limiting dilution analysis; (**e, f**) one-way ANOVA. Data were presented as mean ± standard deviation. COMBO for combination. ns for not significant. Source data are provided as a Source Data file. Illustration for (**c**) was created using BioRender.com.

Having established the importance of the ADAR1-mediated A-to-I RNA editing of *SCD1* resulting in its altered mRNA stability and protein expression, and subsequently 5FU + CDDP chemoresistance and stemness, we then exploited strategies to intervene with this pathway. Treatment of all GC-resistant organoid cells with the SCD1 inhibitor SSI4^23^ would sensitize the cells to 5FU + CDDP chemotherapy (Fig. 5a, Supplementary Fig. 17a) and reduce tumor-initiating cell frequency (Fig. 5b, Supplementary Fig. 17b). We also extended into a proof-of-principle tumor xenograft model whereby GC parental and GC resistant organoids were injected subcutaneously into immunocompromised mice and treated with DMSO or 5FU + CDDP and vehicle or SCD1 inhibitor SSI4 (Fig. 5c). While both the SSI4 treatment group and SSI4 + 5FU + CDDP combination treatment group resulted in a similar reduction in tumor volume, tumor weight and relative tumor growth (Fig. 5d-f), we did note that the tumor-initiating cell frequency which marks self-renewal was significantly more abrogated in the combination group as compared to the SSI4 treatment group alone (Fig. 5g). These suggested that therapeutic targeting of SCD1 may serve as a strategy to overcome 5FU + CDDP resistant in GC intestinal tumors while effectively diminishing the tumor-initiating cell subset.

### SCD1 drives lipid droplets to promote chemoresistance

**Fig. 6:**
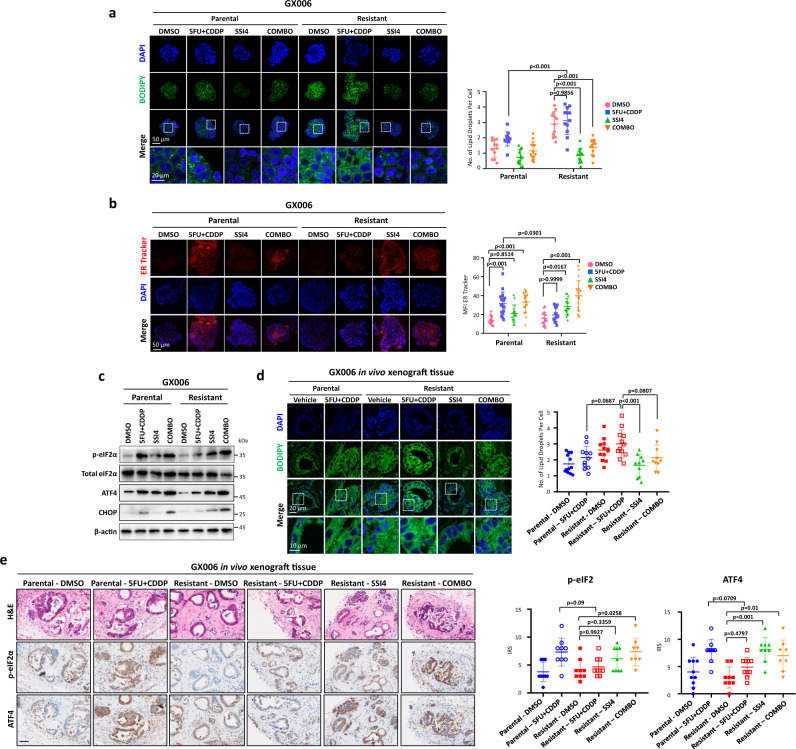
SCD1-driven lipid droplets alleviate chemotherapy-induced ER stress. **a** Representative immunofluorescence images and quantification of BODIPY staining of lipid droplet in parental and resistant GX006 GC organoids treated with DMSO, 5FU + CDDP, SSI4 and combination of 5FU + CDDP and SSI4 (COMBO). Scale bar, 50 μm (low magnification) and 20 μm (high magnification). **b** Representative immunofluorescence images and quantification of ER tracker staining in the indicated treatment groups in GX006 parental and GX006 5FU + CDDP resistant organoids. Scale bar, 50 μm. **c** Western blot analysis for expression of ER stress markers including total and phosphorylated eIF2α, ATF4, and CHOP upon indicated treatment groups in GX006 parental and GX006 5FU + CDDP resistant organoids. β-actin is used as loading control. **d** Representative immunofluorescence images and quantification of BODIPY staining of lipid droplet in the indicated treatment groups in GX006 parental and GX006 5FU + CDDP resistant xenografts. **e** Haematoxylin and eosin (H&E) and IHC staining for phosphorylated eIF2α and ATF4 in the indicated treatment groups in GX006 parental and GX006 5FU + CDDP resistant xenografts. Scale bar, 100μm. (**a**) *n* = 4 independent experiments; (**b**) *n* = 3 independent experiments. (**c**) *n* = 3 independent experiments; (**d**) *n* = 10-12 randomly captured field of view. Parental-DMSO, Resistant-DMSO, Resistant-5FU + CDDP, *n* = 12 images; Parental-5FU + CDDP, *n* = 11 images; Resistant-SSI4 and Resistant-COMBO, *n* = 10 images. (**e**) *n* = 8-10 randomly captured field of view. For p-eIF2α, Parental-DMSO, Parental-5FU + CDDP, Resistant-DMSO, Resistant-5FU + CDDP, Resistant-SSI4, *n* = 9 images; Resistant-COMBO*, n* = 8 images. For ATF4, Parental-DMSO and Resistant-5FU + CDDP, *n* = 10 images; Parental-5FU + CDDP, Resistant-DMSO and Resistant-SSI4, *n* = 9 images; Resistant-COMBO, *n* = 8 images. Significance were calculated by (**a, b**) two-way ANOVA; (**d, e**) one-way ANOVA. Data was presented as mean ± standard deviation. COMBO for combination, IRS for immunoreactive score, MFI for mean fluorescence intensity. ns for not significant. Source data are provided as a Source Data file.

To explore how a dysregulated lipid network confers chemoresistance, we visualized the lipids in the organoids using BODIPY staining and discovered increased lipid droplet, a functional marker of chemoresistance^24^, in the resistant GC organoid lines (Supplementary Fig. 18a). In accordance with previous studies^25^, SCD1 inhibition, either via genetic knockdown or pharmacological means, resulted in a significant reduction of lipid droplets (Fig. 6a, Supplementary Fig. 18b-c and 19). As an adaptive response to chemotherapy-induced ER stress, lipid droplets alleviate ER stress via modulating lipid homeostasis and sequestering misfolded proteins^26,27^. To characterize the ER stress level upon chemotherapy treatment, Western blot was used to detect established ER stress markers, including p-eIF2α, ATF4 and CHOP. Furthermore, the enlargement of ER lumen, a feature of ER stress, was also detected using ER tracker. Our data demonstrated that the resistant GC organoid lines had enhanced ability to suppress ER stress upon chemotherapy treatment as opposed to the parental GC organoid lines, which had elevated ER stress following treatment with chemotherapy (Fig. 6b-c). Strikingly, SCD1 inhibition led to a marked increase in ER stress in the resistant GC line, implying that chemoresistance may be dependent on SCD1-driven lipid droplet formation. BODIPY staining of the xenograft section revealed that resistant GC organoid xenograft harboured a significantly higher level of lipid droplets compared to the parental GC organoid xenograft when treated with chemotherapy (Fig. 6d). Recapitulating the in vitro results, the addition of SSI4 to chemotherapy suppressed lipid droplet formation in the resistant GC organoid xenografts to induce ER stress (Fig. 6d). Additionally, upon treatment with chemotherapy, the parental GC organoid xenografts displayed increased ER stress as indicated by strong p-eIF2α and ATF4 staining as opposed to the low level observed in resistant GC organoid xenografts (Fig. 6e). However, SSI4 treatment could induce ER stress in resistant GC organoid xenografts (Fig. 6e). To confirm the role of lipid droplets in conferring resistance to 5FU + CDDP, we inhibited lipid droplet formation using A922500, which inhibits key lipid droplet formation enzyme diacylglycerol O-Acyltransferase 1 (DGAT1). The successful inhibition of lipid droplet formation by A922500 was confirmed by immunofluorescence (Supplementary Fig. 20a). Inhibition of lipid droplet formation alone did not induce apoptosis but increased apoptosis and ER stress when combined with 5FU + CDDP (Supplementary Fig. 20b–d). Taken together, our results indicated that SCD1-driven lipid droplets suppress chemotherapy-induced ER stress, thereby facilitating tumor survival during the course of chemotherapy treatment.

### SCD1 promotes Wnt/β-catenin signaling to augment cancer stemness

**Fig. 7:**
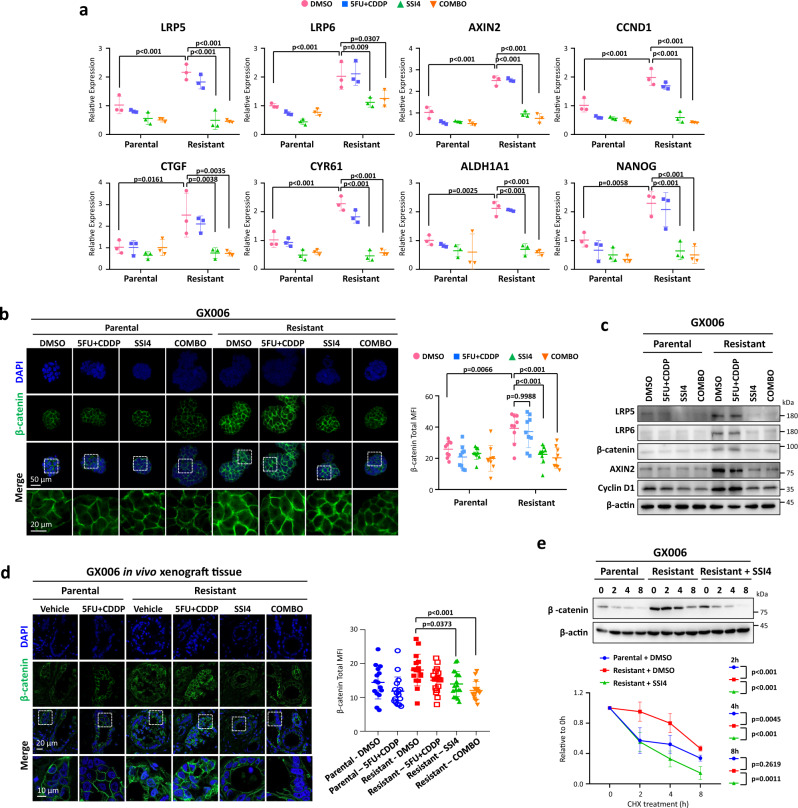
SCD1 drives cancer stemness via augment Wnt/β-catenin signaling. **a** qPCR analysis of *LRP5*, *LRP6*, *AXIN2*, *CCND1*, *CTGF*, *CYR61*, *ALDH1A1* and *NANOG* in the indicated treatment groups in GX006 parental and GX006 5FU + CDDP resistant organoids. **b** Representative immunofluorescence images and quantification of β-catenin staining in the indicated treatment groups in GX006 parental and GX006 5FU + CDDP resistant organoids. Scale bar, 50μm (low magnification) and 20μm (high magnification). **c** Western blot analysis for expression of LRP5, LRP6, β-catenin, AXIN2, cyclin D1 in the indicated treatment groups in GX006 parental and GX006 5FU + CDDP resistant organoids. β-actin is used as the loading control. **d** Representative immunofluorescence images of GX006 parental and GX006 5FU + CDDP resistant xenografts stained with β-catenin. Scale bar, 20μm (low magnification) and 10μm (high magnification). **e** Western blot for β-catenin following cycloheximide (CHX) treatment for 0, 2, 4 and 8 hours in GX006 parental, GX006 5FU + CDDP resistant organoids and GX006 5FU + CDDP resistant organoids treated with SSI4. (**a, c, e)** *n* = 3 independent experiments; (**b**) *n* = 3 independent experiments. (**d**) *n* = 15–19 randomly captured field of view. Parental-DMSO and Resistant-COMBO, *n* = 15 images; Parental-5FU + CDDP and Resistant-DMSO, *n* = 17 images; Resistant-5FU + CDDP, *n* = 16 images; Resistant-SSI4 and, *n* = 19 images. Significance were calculated by (**a, b**) two-way ANOVA; (**d, e**) one-way ANOVA. All data were presented as mean ± standard deviation. COMBO for combination, MFI for mean fluorescence intensity. ns for not significant. Source data are provided as a Source Data file.

SCD1 has been shown to enhance cancer stemness gene expressions^28–30^. In congruence with previous research, inhibition of SCD1 impeded stemness gene expressions (Fig. 7a). Wnt signaling may be impaired upon SSI4 inhibition as a previous study demonstrated that pharmacological inhibition of SCD1 reduces mRNA stability of *LRP5* and *LRP6* that functions as Wnt ligand co-receptors, thereby leading to reduced LRP5/6 protein abundance and Wnt signaling^31^. Consistently, we observed that with the higher SCD1 expression in the resistant GC organoid lines, higher protein level of LRP5/6, β-catenin and Wnt/β-catenin targets, Axin2 and cyclin D1, were observed in the resistant as compared to parental lines (Fig. 7b-c), possibly yielding a greater self-renewal ability (Fig. 1c). Consistently, inhibition of SCD1 led to a reduction in protein level of LRP5/6 β-catenin and Wnt/β-catenin targets (Fig. 7b-c). β-catenin staining of the xenograft section reflected the in vitro observations as compared to the parental GC xenograft, with the resistant GC xenografts showing greater β-catenin level, whereas addition of SSI4 ablated β-catenin (Fig. 7d). As β-catenin is readily degraded by the binding of degradation complex, we also explored if this would affect β-catenin status in the resistant organoids. Through inhibition of protein synthesis by cycloheximide (CHX), we observed increased stability of β-catenin in resistant organoids compared to parental counterpart, while inhibition of SCD1 or knockdown of ADAR1 in resistant organoids reduced its stability (Fig. 7e, Supplementary Fig. 21).

### Overexpression of ADAR1 and hyper-editing/overexpression of SCD1 is strongly associated with the pathogenesis of gastric cancer

**Fig. 8:**
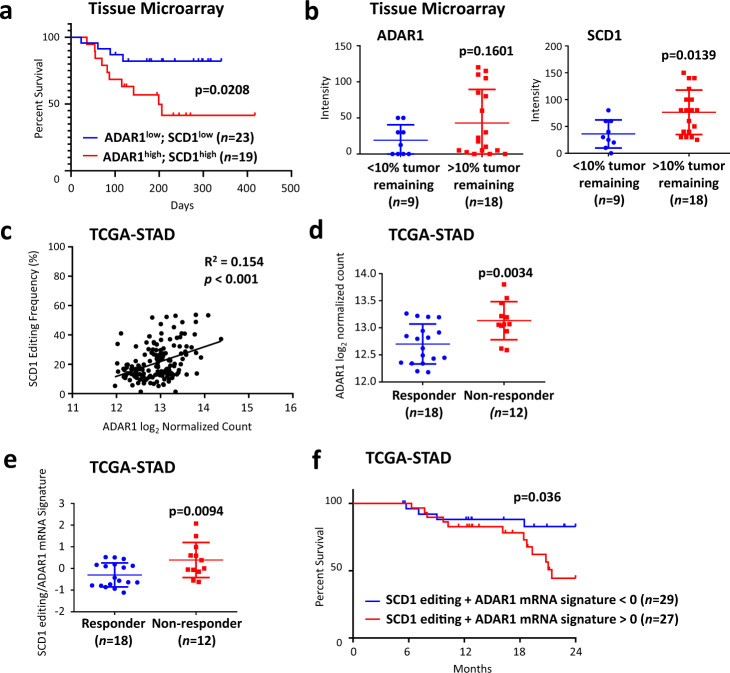
Overexpression of ADAR1 and hyper-editing/overexpression of SCD1 are strongly associated with the pathogenesis of gastric cancer. **a** Kaplan-Meier overall survival plot comparing gastric cancer patients with high ADAR1 and high SCD1 proteomic expression versus low ADAR1 and low SCD1 proteomic expression. All gastric cancer patients were treated with a combination of 5FU and platinum-based chemotherapy in an adjuvant clinical setting. **b** Expression level of ADAR1 and SCD1 and its correlation with percent tumor remaining. All gastric cancer patients were treated with a combination of 5FU and platinum-based chemotherapy in a neoadjuvant clinical setting. **c** Pearson correlation analysis of *ADAR1* mRNA expression with *SCD1* editing in TCGA-STAD. **d** *ADAR1* mRNA expression in responder versus non-responder to chemotherapy treatment in TCGA-STAD. **e** *ADAR1* mRNA/*SCD1* editing signature in responder versus non-responder to chemotherapy in TCGA-STAD. **f** Kaplan-Meier overall survival plot comparing patients with *ADAR1* mRNA/*SCD1* editing signature >0 with *ADAR1* mRNA/*SCD1* editing signature <0. For panels (**d-f**), only GC patients of intestinal subtype treated with 5FU or platinum-based chemotherapy were considered. Significance were calculated by (**a, f**) log-rank test; (**b, d, e**) unpaired two-tailed student t-test; (**c**) two-tailed Pearson **c**orrelation analysis. All data was presented as mean ± standard deviation. Source data are provided as a Source Data file.

To examine the clinical relevance of ADAR1/SCD1 axis in mediating chemoresistance, we examined the level of ADAR1 and SCD1 in patients treated with 5FU- and platinum-based doublet chemotherapy. In a cohort using 5FU- and platinum-based doublet chemotherapy as adjuvant therapy, we stratified patients based on ADAR1/SCD1 expression and found that patients with concomitant high expression of ADAR1 and SCD1 corresponded to worse prognosis (Fig. 8a, Supplementary Fig. 22). In addition, when 5FU- and platinum-based doublet chemotherapy was used as neoadjuvant therapy, high ADAR1 and SCD1 expressions predicted a worse treatment outcome (Fig. 8b). These together suggested that ADAR1/SCD1 may represent a potential prognosis marker for chemotherapy. To substantiate our findings, we explored the GC samples from the TCGA cohort. ADAR1 expression was shown to be highly correlated with *SCD1* editing level (Fig. 8c). Moreover, among patients of intestinal subtype treated with either 5FU-based or platinum-based chemotherapy, non-responders exhibited higher ADAR1 expression compared to responders (Fig. 8d). In addition, we calculated a signature score based on *ADAR1* mRNA expression and *SCD1* editing levels. We showed that non-responders manifested greater signature score compared to responders (Fig. 8e). Similarly, patients with higher signature score had worse prognosis (Fig. 8f).

**Fig. 9:**
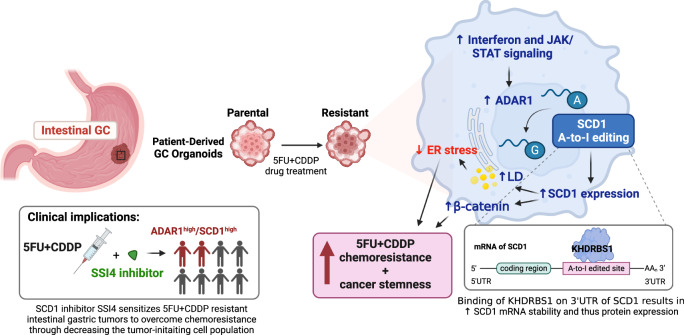
Proposed model for chemoresistance driven by ADAR1-upregulated SCD1 in gastric cancer. Illustration was created using BioRender.com.

Collectively, our work illuminates the mechanism endowing chemoresistance in gastric cancer. These data demonstrate that ADAR1-mediated RNA-editing upregulates SCD1 protein abundance, thereby facilitating lipid droplet formation and β-catenin stability to confer chemoresistance and cancer stemness, leading to survival under chemotherapy-induced stress. Therapeutically, the addition of SCD1 inhibitor to the chemotherapy regimen may augment the treatment efficacy (Fig. 9).

## Discussion

The occurrence of acquired chemoresistance remains a conundrum in achieving durable clinical benefits^32^. Despite substantial effort to decipher chemoresistance, the clinical implication remains rudimentary, partly attributed to the majority of experiments were conducted with the lack of clinically relevant doublet chemotherapy as well as physiologically accurate models. Furthermore, the vast heterogeneity of gastric cancer necessitates the need to administer treatment based on subtype. Here, using organoid models that faithfully recapitulate patient physiological conditions^17^, we established intestinal GC organoid resistant to 5FU + CDDP combination treatment. Notably, the resistant lines depicted augmented self-renewal ability, a trait of significant importance to the relapse post-treatment. While it has been shown that increased mutation burden and genomic alterations, for instance, *MDM2* and *MYC* amplification may influence chemotherapy treatment response^33^, whole-exome sequencing of our model did not display notable consistent changes across the three resistant GC organoid lines. Transcriptome analysis revealed the enrichment of JAK/STAT in the resistant line, corroborating with studies demonstrating JAK/STAT role in treatment resistance and cancer stemness^34,35^, suggesting a transcriptomics or epitranscriptomics aberrations instead of genomic alterations endowed chemoresistance.

Mounting evidence illuminates ADAR1 as a downstream effector of JAK/STAT to augment malignant properties^14,16^. While it has been established that ADAR1 can drive cancer progression, limited study has investigated whether ADAR1 contributes to chemotherapy resistance. Of note, ADAR1 is functionally depicted to promote GC in an RNA editing-dependent manner^11^. Here, our work demonstrated that the upregulation of ADAR1 confers chemoresistance and cancer stemness to the resistant GC organoids. Of significance, only the wild type but not the catalytically-dead mutant can endow chemoresistance to the parental line, highlighting editing activity of ADAR1 is critical to its oncogenic influence. ADAR1 exists in two isoforms, p150 and p110, and there are reports that they have different RNA target due to their preferential localization in the cell. Previous work showed that only p150 is inducible by interferon while here we showed that expression of both p110 and p150 isoforms increased following interferon treatment and in the resistant organoids (Fig. 1g). A recent report pointed to the possibility of downstream translation initiation on p150 isoform to create p110 isoform^36^. Despite showing a slight effect of p150 ADAR1 against 5FU + CDDP treatment, p110 ADAR1 exists at a much higher level than p110 ADAR1 and a stronger effect against 5FU + CDDP treatment in our GC organoids. Thus, we believe that the p110 ADAR1 is the predominant factor contributing towards resistance to chemotherapy in intestinal subtype gastric cancer.

An important function of ADAR1 in normal homeostasis resides on its ability to enrich post-transcriptome diversity^10^. The abundance of editing sites in the human genome allows ADAR1 to govern cancer in a plethora of ways. Given the importance of ADAR1 in normal homeostasis^37^, targeting ADAR1 may result in severe side effects. Conceivably, identifying and targeting oncogenic hyper-edited genes downstream of ADAR1 may be more therapeutically viable. To this end, we leveraged WES coupled with RNA-seq to identify putative hyper-edited genes. Our data demonstrated that the resistant GC organoids harboured a markedly enriched RNA editome. Particularly, we noticed an enrichment of hyper-edited genes involved in lipid metabolism. As aforementioned, GO analysis of our in-house RNA-seq revealed an altered lipid metabolism in the resistant GC organoid lines. Taken together, our data illustrate that ADAR1 may facilitate a dysregulated lipid network to drive chemoresistance in an RNA editing-dependent manner. Accordingly, we focus on SCD1 due to its targetability and association with a worse prognosis. RNA editing in the 3’UTR may lead to changes in protein expression via modulating the miRNA binding sites, mRNA stability and localization. Our data depicted that ADAR-edited 3’UTR of *SCD1* has a greater propensity to be bound by KHDRBS1, consequently, a greater mRNA stability and protein expression.

We discovered a regulation pathway of SCD1 through A-to-I editing by ADAR1 and binding by KHDRBS1. There are many mechanisms by which protein expression is altered through post-transcriptional modification, including transcription activity, RNA stability and translation efficiency. In this study, we excluded the possibility of transcription through measuring mRNA expression and confirmed the change in stability by Actinomycin D treatment and luciferase reporter assay. However, we did not assess the effect of A-to-I editing on translational efficiency. The link between translation efficiency and RNA stability is complex. For example, translation has been shown to couple with RNA decay and that increasing translation, as determined by the increase in polysome to monosome ratio, showed a negative correlation with RNA stability^38,39^. These suggest that increase in RNA stability may be the main reason for the increase in protein level rather than a simultaneous increase in RNA stability and translation efficiency. The use of polysome profiling or Ribo-seq in future study will provide valuable information on connecting A-to-I editing with RNA translation efficiency.

Compiling experiments illustrate alterations in lipid network fuel drug resistance and self-renewal^40,41^. Indeed, our work showed either genetic manipulation or pharmacological inhibition of SCD1 suppressed chemoresistance and cancer stemness, supporting SCD1 as an important mediator in malignant progression^42–44^. Previous studies on SCD1 in gastric cancer is scarce, with only two reports documenting its correlation with advanced cancer stage and tumorigenic role through EMT and Hippo/YAP pathway^29,45^. Here, we identified two pathways by which SCD1 can affect gastric cancer, alleviation of ER stress and activation of β-catenin signaling. In line with previous studies^31^, inhibition of SCD1 impeded LRP5/6, leading to a reduction in cancer stemness genes expression. Strikingly, collateral sensitivity, a feature deemed as the elevated vulnerability of resistant cells to a second therapeutic agent^46^, was evident in the resistant GC organoid lines when treated with SCD1 inhibitor, posing SCD1 inhibition as an exploitable molecular vulnerability to circumvent chemoresistance and cancer stemness.

With regards to clinical relevance, our data shed light on the therapeutic potential of ADAR1/SCD1 axis as a prognostic marker. Fluoropyrimidine and platinum-based drugs remain a commonly used combination chemotherapy for gastric cancer patients. However, despite initial response, patients will eventually develop resistance to this combination therapy and lead to poor prognosis. Here, we discovered a previously unrecognized mechanism by which ADAR1 is upregulated in gastric cancer organoids to develop resistance towards 5FU + CDDP through increase editing and thereby protein level of SCD1. ADAR1/SCD1 protein high expression corresponds to markedly worse prognosis in patients treated with 5FU- and platinum-based doublet regimen. Capitalizing on the data from TCGA-STAD cohort, we demonstrated that the *ADAR1* expression/*SCD1* editing signature predicts chemotherapy treatment outcome as well as worse overall survival. It is noteworthy that a study conducted by the "3G" trial demonstrated that high editing corresponds to a better prognosis in patients treated with chemotherapy^12^. However, the signature score in this study was based on the preselection of editing sites positively correlated with prognosis. In addition, the study included all the subtypes of gastric cancer and was not reflecting the response of a particular subtype. Furthermore, we explored the mechanism of acquired resistance towards fluorouracil and platinum-based doublet chemotherapy while the paper focused on primary response. It will be interesting to extend our study to investigate the contribution of *SCD1* editing in predicting primary response to doublet chemotherapy, given the promising correlation shown from the TCGA cohort.

Collectively, our conglomerate effort encompassing organoid platform, molecular profiling and in vitro and in vivo functional assays discovered ADAR1/SCD1 axis governs chemoresistance by enhancing lipid droplet formation to neutralize ER stress induced by chemotherapy. Additionally, our study illuminates the potential clinical implication of SCD1 inhibition. Given that our work focuses on delineating the chemoresistance in intestinal subtype, whether the ADAR1/SCD1 axis contributes to chemoresistance in other subtypes warrants further investigations.

## Methods

### Study approval

Clinical samples for tissue microarray and IHC were obtained by Prof. Jing-Ping Yun at the Sun Yat-sen University Cancer Centre in Guangzhou, China, with the approval by the Institutional Review Board for ethical review from the University (SL-B2023-095-01). The procurement of all clinical information has received consent from patients. The approval for establishing patient-derived organoid models was obtained from Institutional Review Board of the University of Hong Kong and the Hospital Authority Hong Kong West Cluster (IRB reference ID: UW14-257). Participants gave informed consent to participate in the study. License to conduct experiments on animals was obtained from Department of Health, Hong Kong SAR. Approval to conduct animal work at the University of Hong Kong was obtained from the Committee on the Use of Live Animals in Teaching and Research at the University of Hong Kong.

### GC organoid culture and drug resistance training

GC organoids used in the current study were established in our laboratory and previously characterized in detail^17^. For the establishment of GC organoids resistance to 5-fluorouracil (Sigma-Aldrich) and cisplatin (Cayman) treatment, organoids were cultured with IC_10_ initially with the dose increased gradually until reaching IC_50_ or at dose which organoids remained alive but did not proliferate.

### Cell line culture

MKN28 (JCRB0253) and NUGC3 (JCRB0822) were purchased from JCRB Cell Bank and grown in RPMI medium supplemented with 10% FBS, 1% Penicillin/Streptomycin. 293T/17 was purchased from ATCC and grown in DMEM supplemented with 10% FBS, 1% penicillin/streptomycin, 1x GlutaMAX^TM^.

### Bioinformatics analysis

A list of bioinformatic analysis tools used in this study are summarised in Supplementary Table 1 with detailed parameters available on request. For WES and RNA-seq, analysis pipelines for variant calling, DNA copy number analysis and gene expression analysis were same as previously reported^17^.

### Identification of A-to-I editing in RNA

Potential A-to-I editing sites were identified as A to G (or T to C on the complement strand) mutation in the RNA with read depth > 7 and allele depth > 2 (*n* = 37,140). Sites with variants found in the DNA of corresponding GC organoids were removed (*n* = 31,773). The sites were then annotated using data downloaded from the REDIportal^47^. Sites absence in A-to-I editing databases [RADAR^48^ and DARNED^49^] and not edited in the stomach as defined by the inosinome Atlas^50^ were removed (*n* = 6025). For each site, the percentage of reads containing “G” or “C”, as alternative from “A” or “T” respectively, was calculated and the difference between the paired two lines were computed (resistance relative to parental). To be considered a hyper-edited site, that site must have at least 10% change in two of the three organoid lines examined (*n* = 711). Genes with edited sites were then grouped based on biological process information from the Protein Atlas with aid from the Entrez database. For validation of A-to-I editing of SCD1, region of interest was amplified by PCR and confirmed by Sanger sequencing using primers listed in Supplementary Table 2.

### RNA extraction, cDNA synthesis and qPCR

Total RNA was extracted using RNA-IsoPlus (Takara) and cDNA was synthesized by PrimeScript RT Master Mix (Takara). qPCR was performed with EvaGreen qPCR master mix (ABM) and primers listed in Supplementary Table 2 on a LightCycler 480 II analyser (Roche) with data analysed using the LightCycler 480 II software (Roche). Relative expression differences were calculated using the 2^-ΔΔCt^ method.

### Transcriptome sequencing (RNA-seq) and whole-exome sequencing (WES) of organoids

GC organoids were collected using cell recovery solution (Corning). After media removal, 500 μL cell recovery solution was added to each well to collect the organoids. The organoids were left in solution at 4 °C for 30 min to recover the organoids from Matrigel. After that, organoids were centrifuged at 300 g for 5 min, washed with PBS once, and stored in −80 °C until extraction. DNA and RNA were extracted from organoids using AllPrep DNA/RNA/miRNA Universal Kit (Qiagen) as per protocol. RNA-seq samples were subjected to cDNA library construction using the KAPA Stranded mRNA-seq Kit (KR0960-v3.15). Libraries were then sequenced using the PE101 HiSeq1500 or PE151 NovaSeq6000. For WES, 550 ng of genomic DNA were input for library preparation after fragmentation by Covaris S2, following the KAPA Hyper Prep Kit (KR0961-V1.14) protocols, with selection for a library size range of 250–450 bp. 300 ng per library DNA each from 12 samples were normalized and combined into a single pool for exome capture using the xGen® Lockdown® Probes and Reagents based on their standard protocols. Captured libraries were sequenced using the HiSeq1500 with paired end 101 bp reads or NovaSeq6000 with paired-end 151 bp reads. The mean sequencing depth for the organoids and germline DNA were approximately 50X.

### Correlation analysis of copy number variation

To statistically test the alterations in copy number between samples, the correlations of log R ratio (LRR) at each window of SNP markers between paired parental and resistant organoids was computed.

### Public data analysis (TCGA-STAD)

RNA-seq data (fastq files) of 161 gastric cancer samples of intestinal subtype from TCGA were downloaded from the dbGaP repository, under accession phs000178.v11.p8. A bioinformatics pipeline adapted from a previously published method^48^ was employed to identify RNA editing events as described before^51^. Raw fastq reads were mapped to the reference human genome (hg19) and a splicing junction database generated derived from transcript annotations of University of California Santa Cruz, RefSeq, Ensembl, and GENCODE (v19) using BWA with default parameters (BWA-MEM algorithm, v0.7.17-r1198)^52^. To obtain reliable results, the reads with mapping quality score <20 were removed, and PCR duplicates were discarded with Samtools (rmdup function, v1.9)^53^. Junction-mapped reads were then converted back to the genomic-based coordinates. An in-house Perl script was used for variant calling from Samtools pileup data, and the sites with at least two supporting reads were kept. Next, the candidate events were filtered by excluding the single-nucleotide polymorphisms (SNPs) reported in different cohorts [1000 Genomes Project^54^ and NHLBI GO Exome Sequencing Project (http://evs.gs.washington.edu/EVS/), and removing the sites within the first six bases of the reads caused by imperfect priming of random hexamer when synthesizing cDNA. For the sites not located at Alu element regions, the candidate sites within the four bases of a splice junction on the intronic side and those residing in the homopolymeric regions and in the simple repeats were all excluded. Finally, candidate variants located in the reads that align to the non-unique regions of the genome by the BLAST-like alignment tool^55^ were also removed.

### Calculation of SCD1 editing + ADAR1 mRNA signature

The *z*-score of ADAR1 mRNA expression and SCD editing level were calculated for each patient from the TCGA-STAD cohort. The signature score was then derived from the average of *z*-score of ADAR1 mRNA expression and *z*-score of SCD editing level for each patient.

### Plasmids and lentivirus transfection

Plasmids for overexpression of wild-type (WT) ADAR1 and enzymatically dead mutant (MUT) ADAR1 that contains point mutations in its catalytic site (H910Y and E912A) were provided by Dr Leilei Chen (National University of Singapore). Plasmid for overexpression of p150 ADAR1 isoform was prepared by cloning CDS of p150 ADAR1 isoform into pLenti CMV Blast DEST (706-1) vector (Addgene #17451). Plasmids for the knockdown of ADAR1 expression by shRNA were cloned using pLKO.1-blast lentiviral backbone vector (Addgene #26655). The shRNA sequences were cloned into the EcoRI and AgeI site with the following shRNA sequences: NTC (CCGGCAACAAGATGAAGAGCACAACTCGAGTTGGTGCTCTTCATCTTGTTGTTTTTG), ADAR1 KD1 (CCGGGACTGCGAAGGATAGTATATTCTCGAGAATATACTATCCTTCGCAGTCTTTTTG), ADAR1 KD2 (CCGGAGTTTCCTGCTTAAGCAAATACTGCAGTATTTGCTTAAGCAGGAAACTTTTTTG). Plasmids for knockdown of SCD1, STAT3 and KHDRBS1 expression by shRNA were cloned using pLKO.1-puro lentiviral backbone vector (Addgene # 8453). The shRNA sequences were cloned into the EcoRI and AgeI site with the following shRNA sequences: SCD1 KD1 (CCGGCTACGGCTCTTTCTGATCATTCTCGAGAATGATCAGAAAGAGCCGTAGTTTTTG), SCD1 KD2 (CCGGCCCACCTACAAGGATAAGGAACTCGAGTTCCTTATCCTTGTAGGTGGGTTTTTG), STAT3 KD1 (CCGGCCTGAGTTGAATTATCAGCTTCTCGAGAAGCTGATAATTCAACTCAGGTTTTTG), STAT3 KD2 (CCGGGCAAAGAATCACATGCCACTTCTCGAGAAGTGGCATGTGATTCTTTGCTTTTTG), KHDRBS1 KD1 (CCGGGTTATGAGCAAACTTGTTACTCTCGAGAGTAACAAGTTTGCTCATAACTTTTTG), KHDRBS1 KD2 (CCGGGATGAGGAGAATTACTTGGATTCTCGAGATCCAAGTAATTCTCCTCATCTTTTTG). 293T/17 cells (Invitrogen) were used for lentivirus preparation. Overexpression or shRNA knockdown plasmids were transfected with 3^rd^ generation lentivirus packaging plasmids using polyethyleneimine (Sigma-Aldrich). 16 h post-transfection, media with plasmids were removed and replenished with complete cell culture media. After 72 h incubation, lentivirus was collected, concentrated by ultracentrifugation at 25,000 rpm for 2 h and resuspended with complete organoid growth media at 4 °C for 1 h. Cells were then mixed with concentrated lentivirus and 0.8 µg/mL polybrene (Sigma-Aldrich), centrifuged at 300 g for 1 h at room temperature and incubated at 37 °C for 6 h. After that, transduced cells were collected by centrifugation and seeded in Matrigel as described in the organoid culture section. Antibiotics selection was started 3 days post-transduction.

### Protein extraction and western blot analysis

Protein lysates were extracted, quantified, resolved and transferred onto a PVDF membrane (Millipore) and probed with primary and secondary antibodies prior to detection using chemiluminescence system (GE Healthcare). The following antibodies were used: ADAR1 (1:1000, Cell Signaling Technology, #14175), ADAR1 p150 specific (1:1000, abcam, ab168809), ADAR2 (1:1000, Sigma-Aldrich, SAB1405426), SCD1 (1:1000, abcam, ab19862), ATF4 (1:1000, Santa Cruz Biotechnology, sc-390063), CHOP (1:500, Cell Signaling Technology, #2895), p-eIF2α (1:1000, Cell Signaling Technology, #9721), total eIF2α (1:1000, Cell Signaling Technology, #9722), p-STAT3 (1:1000, Cell Signaling Technology, #9134), total STAT3 (1:1000, Cell Signaling Technology, #9139), p-JAK2 (1:1000, Cell Signaling Technology, #3771), total JAK2 (1:1000, Cell Signaling Technology, #3230), LRP5 (1:1000, abcam, ab36121), LRP6 (1:1000, abcam, ab134146), β-catenin (1:1000, BD Biosciences, #610153), AXIN2 (1:1000, abcam, ab109307), cyclin D1 (1:1000, Santa Cruz Biotechnology, sc-753), KHDRBS1 (1:1000, abcam, ab86239), β-actin (1:5000, Sigma-Aldrich, A5316), Histone H3 (1:1000, abcam, ab24834), α-Tubulin (1:1000, Sigma-Aldrich, T9026). Images were captured using BioRad ImageLab Touch software (version 2.4.0.03).

### In vitro extreme limiting dilution spheroid formation assay

Dissociated single cells were cultured in medium supplemented with 0.25% methylcellulose (Sigma-Aldrich). Cells were seeded at limiting dilutions in polyHEMA-coated plates, supplemented with fresh media every other day. Spheroid formation was measured 7-10 days after seeding. Tumor-initiating cell frequency was calculated using extreme limiting dilution analysis^56^.

### Annexin V-PI flow cytometry

Cells were treated with chemotherapy and/or inhibitors for 6 days, with the medium changed 3 days after the first treatment. Cells were stained with FITC-conjugated Annexin V and propidium iodide (PI) as provided by the Annexin V-FLUOS staining kit (Roche). Samples were analysed on a BD FACSCanto II (BD Biosciences) or Novocyte Advanteon BVYG (Agilent Technologies) with data analysed by FlowJo (Tree Star).

### CellTiter-Glo cell viability assay

Cells were treated with chemotherapy for 6 days, followed by measurement according to manufacturer’s protocol (Promega).

### In vivo animal studies

All mice were housed in Association for Assessment and Accreditation of Laboratory Animal Care International (AAALAC)-credited facility in 12 hours light/dark cycle (7:00-19:00 light, 19:00-7:00 dark), with controlled room temperature (23 ± 2 °C) and humidity (30-70%), in groups according to stocking density as recommended in the guide. GC organoids and cells were injected subcutaneously into male NOD *Scid* gamma mice (NOD.Cg-*Prkdc*^*scid*^ *IL2rg*^*tm1wjl*^/SzJ0) and male nude mice (BALB/AnN-nu), respectively. Treatment began when tumors reached an average volume of 80 mm^3^. 5FU and CDDP combination was administered via intraperitoneal injection thrice a week for two weeks. SSI4 was administered via oral gavage daily for two weeks. The doses used for 5FU, CDDP and SSI4 were 10 mg/kg, 2 mg/kg and 1 mg/kg, respectively. Tumor volume was monitored thrice a week and calculated as follows: tumor volume = 0.5 x L x W^2^, with L and W, as the largest and smallest diameters, respectively. Animal research ethics was approved by and performed in accordance with the Committee on the Use of Live Animals in Teaching and Research (CULTAR) at the University of Hong Kong. According to the CULTAR guidelines, the diameter of a single tumor should not exceed 15 mm in mice for therapeutic studies. At the endpoint, animals were euthanised using cervical dislocation under anesthesia as approved by CULTAR.

### Immunohistochemistry (IHC)

Formalin-fixed, paraffin-embedded tissue sections were heated in sodium citrate buffer. Endogenous peroxidase activity was inhibited by 3% hydrogen peroxide. Sections were incubated at 4 °C overnight with the following antibody: ADAR1 (1:300, Cell Signaling Technology, 14175), SCD1 (1:300, abcam, ab19862), STAT3 (1:300, Cell Signaling Technology, 9139), p-eIF2α (1:100, Cell Signaling Technology, #9721) and ATF4 (1:100, Santa Cruz Biotechnology, sc-390063). Slides were developed with DAB+ Substrate-Chromogen System (Dako) and counterstained with Mayer’s haematoxylin. Sections were imaged using Vectra Polaris (PerkinElmer).

### GC clinical samples and IHC scoring

Tissue sections harvested from GC patients who underwent 5FU and platinum-based combination treatment were stained for ADAR1 and SCD1 as described in the immunohistochemistry section. A semi-quantitative immunoreactive score (IRS) was performed by considering intensity (0 for negative, 1 for low, 2 for moderate and 3 for strong) as well as the percentage of positively stained cells (0 for 0%, 1 for 1-10%, 2 for 11-50%, 3 for 51-80% and 4 for >80%). Both indexes were multiplied to yield the IRS. ADAR1 and SCD1 expression levels were defined by categorizing the highest and lowest quarters into high and low expression groups, respectively. Patients were then further segregated into ADAR1^high^/SCD1^high^ and ADAR1^low^/SCD1^low^.

### Immunofluorescence imaging of organoids

Organoids were dissociated from Matrigel using cell recovery solution (Corning) as aforementioned. Organoids were then processed as per published protocol^57^. Organoids were stained with the following antibody: ADAR1 (1:100, Cell Signaling Technology, 14175), SCD1 (1:100, abcam, ab19862), and KHDRBS1 (1:1000, abcam, ab86239). Organoids were mounted and counterstained with antifade DAPI (Invitrogen).

### BODIPY 493/503 lipid droplet and ER tracker staining

Cells were incubated with 5 µg/mL BODIPY 493/503 (Invitrogen) or 1 µM ER tracker red (Invitrogen) for 1 h. Cells were fixed with 2% paraformaldehyde, mounted and counterstained with anti-fade DAPI (Invitrogen). Slides were imaged by confocal microscopy (Carl Zeiss) using Zen 3.0 software and quantified by ImageJ software. To quantify lipid droplets, the number of Maxima (point which emits saturated signal) was identified with an arbitrary threshold using ImageJ to determine the number of lipid droplets per image. The number of cells was determined by counting the number of DAPI-marked nucleus. The data is presented as the number of lipid droplet divided by the number of cells. To quantify ER tracker, the mean fluorescence intensity (MFI), which accounts for area and intensity of the target of interest, of the ER tracker is quantified using ImageJ.

### Interferon (IFN) treatment

Organoids were seeded at 50,000 cells per well and cultured for three days prior to interferon treatment. The organoids were treated with 1000 U/ml of universal type I IFN (PBL Assay Science, #11200) for 24 h and collected for western blot analysis.

### Inhibition of STAT3 signaling

Organoids were treated with 2 µM BBI608 (Selleckchem, #S7977) for 24 h and collected for western blot analysis.

### Inhibition of SCD1 and DGAT1

SCD1 inhibitor (SSI4) was provided by John Copland (Mayo Clinic Florida). DGAT1 inhibitor (A922500) was purchased from Sigma-Aldrich (#A1737). The working concentration for SSI4 and A922500 were 1 µM and 10 µM, respectively. Organoids were treated with inhibitors 48 h prior to being processed for immunofluorescence staining (BODIPY and ER tracker). For in vitro flow cytometry apoptosis assay, the organoids were treated with the inhibitors for 6 days, with the medium changed 3 days after the first treatment and processed as aforementioned.

### Subcellular fractionation of organoids

Subcellular fractionation was performed as previously described^58^. Briefly, the organoid cultures were dissociated using TrypLE and homogenized with the hypotonic medium. The lysate was then centrifuged for 10 min at 6300 g and separated into cytoplasmic fraction (supernatant) and nuclear fraction (pellet). The cytoplasmic fraction was transferred to a new Eppendorf. The nuclear fraction was washed with TSE buffer (10 mM Tris, 300 mM sucrose, 1 mM EDTA, 0.1% IGEPAL-CA 630 v/v, pH 7.5) for three times. Both nuclear and cytoplasmic fractions were boiled with 6X loading buffer prior to analysis of protein expression by Western blot.

### Cycloheximide (CHX) treatment

Complete organoid media with DMSO or SSI4 were added to organoid culture 24 h prior to CHX treatment. CHX (Sigma-Aldrich #239764) was mixed into 100 μl of media without supplements and added into the organoid cultures. The working concentration of CHX used was 100 μM. At 0, 2, 4 and 8 h post-CHX treatment, the organoids were collected with cell recovery solution (Corning) as aforementioned. Western blot analysis was carried out to measure the expression of the protein of interest. Expression level were quantified with ImageJ and the stability of protein was presented as relative to time 0 h.

### 3’UTR luciferase reporter assay

3’UTR of *SCD1* was cloned into pMIR-REPORT (Invitrogen) and transfected into cells using the mouse/rat hepatocyte nucleofector kit (Lonza) on the Nucleofector 2b device (Lonza) following manufacturer’s recommendation with modifications. In brief, 5 µg pMIR-REPORT carrying the 3’UTR of *SCD1* and 500 ng pRL Renilla reporter plasmids were used per reaction with program T-20. Cells were then transferred to 10 mL pre-warmed advanced DMEM/F12 and centrifuged at 200 g for 5 min to collect cells. Transfected cells were seeded and luciferase activity was measured by Dual-Glo luciferase assay (Promega) 3 days post-transfection.

### RNA stability measurement

Cells were cultured in complete organoid media without ROCK inhibitor, plus 10 µg/mL actinomycin D (Sigma-Aldrich). At 3, 6 and 24 h post actinomycin D treatment, cells were harvested for *SCD1* mRNA expression analysis by qPCR as described in the qPCR section. Data expressed as percentage remaining against time 0 h.

### Identification of SCD1 RNA binding protein

Binding or RNA binding protein on the 3’UTR of *SCD1* was predicted with RBPmap^21^. Potential binding partners were identified and ranked by *z*-score and *p*-value.

### Prediction of RNA secondary structure by RNAfold

The complete 3’UTR sequence of *SCD1* RNA were used as input for prediction RNA secondary structure using default setting in RNAfold^22^. The minimum free energy (MFE) structure is shown.

### RNA immunoprecipitation (RIP) assay

RIP of KHDRBS1 was conducted using Magna RIP RNA-binding protein immunoprecipitation kit (Merck Millipore), following manufacturer’s protocol. In brief, 1 mg protein was incubated with 2 µg KHDRBS1 antibody (abcam, ab86239). The expression level of A-to-I editing site on 3’UTR of *SCD1* of the pulldown was measured by qPCR as described in the qPCR section. Successful immunoprecipitation of KHDRBS1 was confirmed by western blot analysis.

### Lipidomic analysis

Organoids were collected using cell recovery solution (Corning) and washed twice with ice-cold saline. Samples were then separated into two parts for processing for polar and nonpolar metabolites detection. For non-polar metabolites detection, 100 μL of chloroform with 20 μg C19:0 fatty acid internal standard was spiked to the sample. The sample was homogenized after 2 cycles of sonication at 10 microns for 20 s on ice and 10 s pause time. The sample was centrifuged for 5 min at 16,000 g and at 4 °C. The pellet was separated and stored at −80 °C. The samples were then transesterified by the addition of 1 mL of methanol and 50 μL of concentrated hydrochloric acid (35%, w/w). The solution was overlaid with nitrogen and the tube was tightly closed. After vortexing, the tube was heated at 100 °C for 1 h. Once cooled to room temperature, 1 mL of hexane and 1 mL of water were added for FAMEs extraction. The tube was vortexed and after phase separation, up to 1 μL the hexane phase was injected for GC-MS analysis. GC/MS chromatogram was then acquired in SCAN and SIM mode in an Agilent 7890B GC - Agilent 7010 Triple Quadrapole Mass Spectrometer system (Agilent Technologies). The sample was separated through an Agilent DB-23 capillary column (60 m × 0.25 mm ID, 0.15μm film thickness) under constant pressure at 33.4 psi. For polar metabolites detection, 1 mL of methanol/water (80%, v/v) with 200 ng norvaline internal standard was added to the sample. The sample was homogenized after 2 cycles of sonication at 10 microns for 20 s on ice and 10 s pause time. The sample was centrifuged for 5 min at 16,000 g and at 4 °C. 500 μL supernatant was dried under a gentle stream of nitrogen at room temperature for derivatization. The dried residue was redissolved and derivatized for 2 h at 37 °C in 40 μL of methoxylamine hydrochloride (30 mg/mL in pyridine) followed by trimethylsilylation for 1 h at 37 °C in 70 μL MSTFA with 1% TMCS. Up to 1 μL sample was injected for GC-MS/MS analysis. GC-MS/MS chromatogram was acquired in SCAN and MRM mode in an Agilent 7890B GC - Agilent 7010 Triple Quadrapole Mass Spectrometer system. The sample was separated through an Agilent DB-5MS capillary column (30 m × 0.25 mm ID, 0.25μm film thickness) under constant flow at 1 mL/min. Data analysis was performed using the Agilent MassHunter Workstation Quantitative Analysis Software (version B.07.01 SP1/Build 7.1.524.1).

### Seahorse bioenergetic analysis

Lipid oxidation of cells was measured using Seahorse XF palmitate oxidation stress test kit (Agilent Technologies) as per protocol using software Wave (version 2.6.3.5).

### Statistical analysis

All data were presented as mean ± standard deviation. Two-tailed student’s *t*-test was used for comparison between two independent groups and paired *t*-test was used for paired data. One-way ANOVA with Tukey post-hoc adjustment was used for analysis for more than two groups. In vivo experiments with continuous measurements were analysed with two-way ANOVA with repeated measurement. Comparison with *p* value less than 0.05 was regarded as statistically significant.

### Reporting summary

Further information on research design is available in the Nature Portfolio Reporting Summary linked to this article.

## Supplementary information

Supplementary Information

Reporting Summary

Description of Additional Supplementary Files

Supplementary Data 1

## Data Availability

The transcriptome sequencing data generated in this study has been deposited in the GEO database under accession code GSE220137. The whole-exome sequencing data generated in this study has been deposited in the ENA database under accession code PRJEB60472. The TCGA-STAD publicly available data used in this study are available in the dbGaP repository, under accession phs000178.v11.p8. Curated A-to-I editing sites in RADAR, DARNED and inosinome Atlas are available from http://srv00.recas.ba.infn.it/atlas/index.html. Reference human genome (hg19) and splicing junction annotation database are available from University of California Santa Cruz (http://genome.ucsc.edu/cgi-bin/hgGateway?db=hg19), RefSeq (https://www.ncbi.nlm.nih.gov/refseq/), Ensembl (https://grch37.ensembl.org/index.html), and GENCODE (v19) (https://www.gencodegenes.org/human/release_19.html). The remaining data are available within the Article, Supplementary Information or Source Data file. Source data are provided with this paper.

## Source data

Source Data
