# Supplementary material for: ADAR1-mediated RNA editing of SCD1 drives drug resistance and self-renewal in gastric cancer
Source: Nat Commun. 2023 May 19;14:2861. doi: 10.1038/s41467-023-38581-8 (PMC10199093; doi:10.1038/s41467-023-38581-8)
Supplement: Supplementary file 1 — Supplementary Information [file 41467_2023_38581_MOESM1_ESM.pdf]

Supplementary Information: ADAR1-mediated RNA editing of SCD1 drives drug resistance and self-renewal in gastric cancer

Table of content:

Supplementary Figure 1-22

Supplementary Table 1. List of bioinformatic analysis tools used in this study.

Supplementary Table 2. PCR primers used in this study.

Supplementary Data 1. List of hyperedited A-to-I editing sites found in 5FU+CDDP resistant GC organoids.

Supplementary References

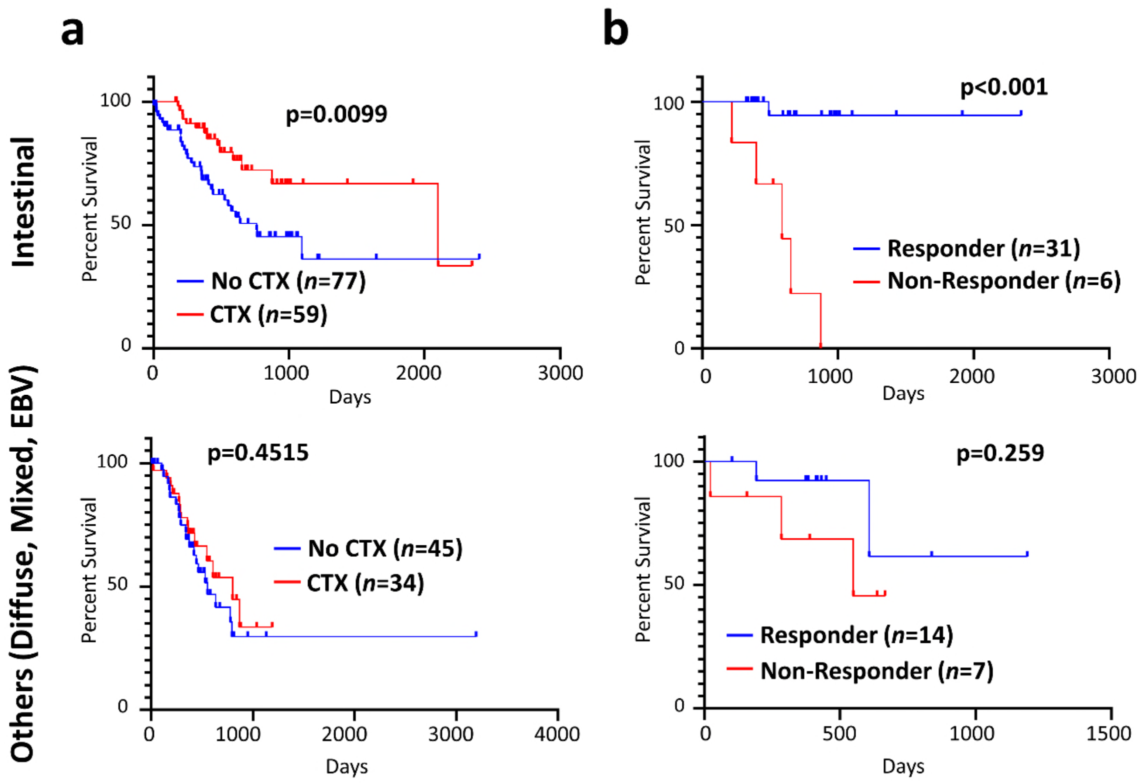

Supplementary Figure 1. a Kaplan-Meier plot comparing overall survival of gastric cancer patients of intestinal subtypes (top) and other subtypes (bottom) treated with chemotherapy (CTX) versus patients treated without chemotherapy (no CTX) in the TCGA-STAD cohort. b Kaplan-Meier plot comparing overall survival of gastric cancer patients of intestinal subtypes (top) and other subtypes (bottom) that are responsive to chemotherapy versus patients that are not responsive to chemotherapy in the TCGA-STAD cohort. For all panels, significance were calculated by log-rank test. CTX for chemotherapy. Source data are provided as a Source Data file.

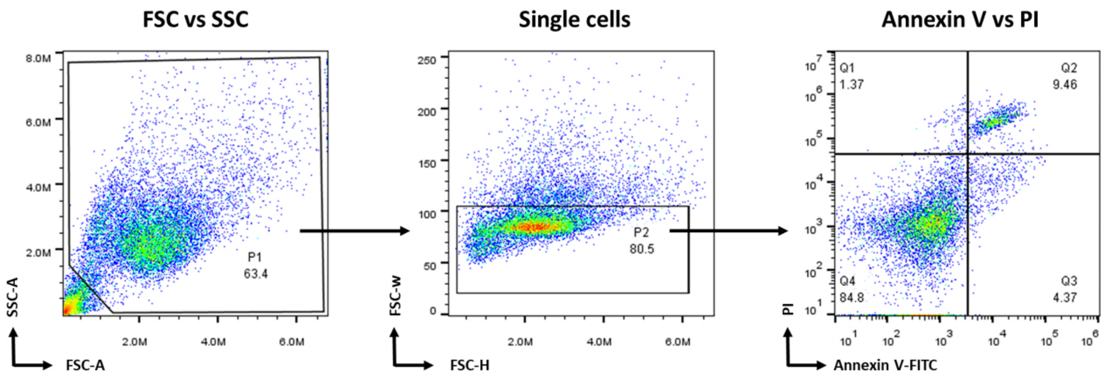

Supplementary Figure 2. Representative gating strategy for Annexin V-PI flow cytometry analysis. FSC for forward scatter, SSC for sideward scatter, PI for propidium iodide.

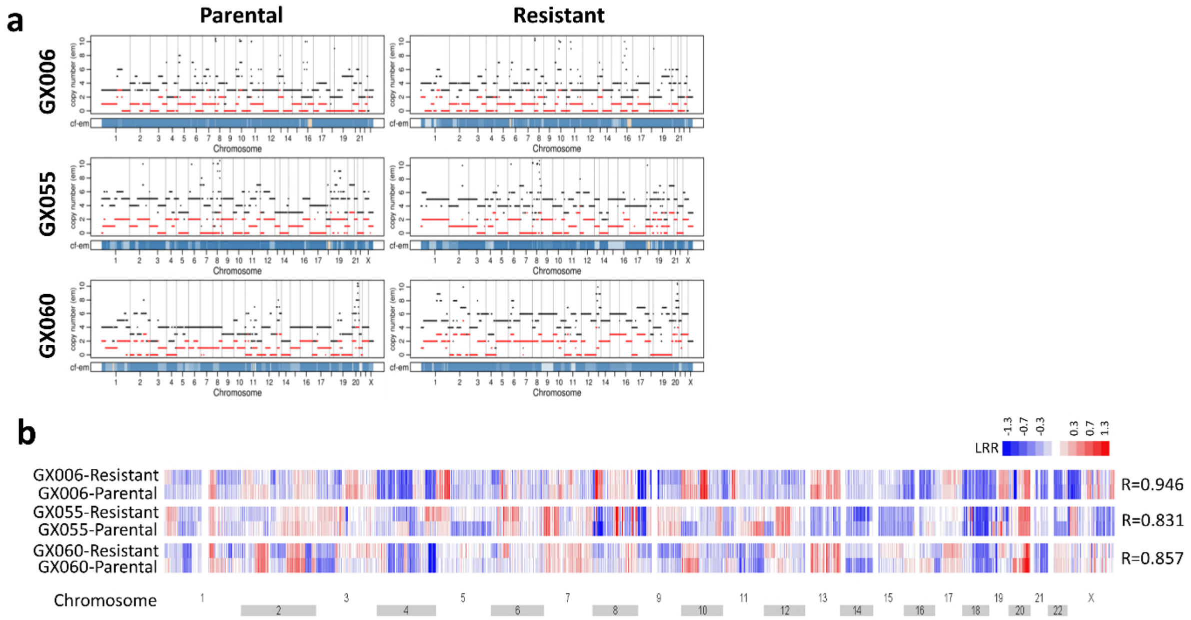

Supplementary Figure 3. a Overview of genomic alterations identified by whole-exome sequencing of DNA extracted from three pair of parental versus 5FU+CDDP resistant organoid lines. b Correlation of log R ratio (LRR) between paired parental and resistant organoid lines.

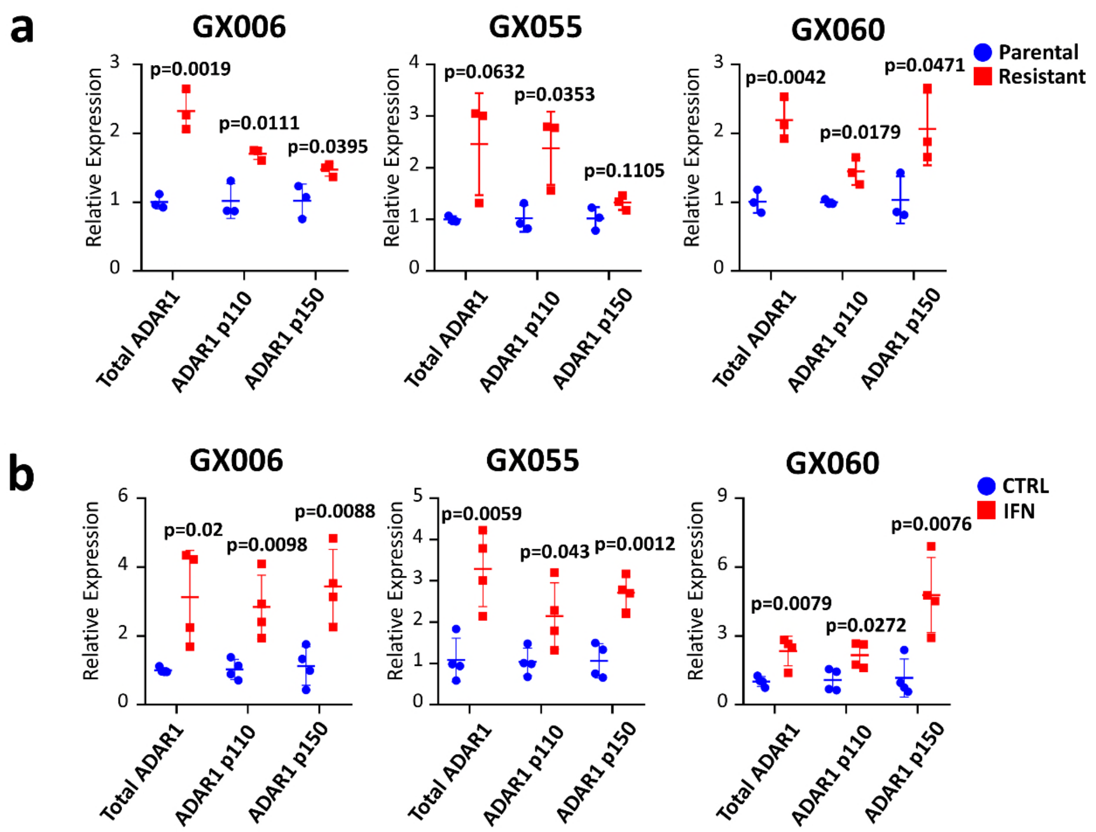

Supplementary Figure 4. a, b qPCR analysis of total ADAR1, ADAR1 p110 isoform and ADAR1 p150 isoform in parental and paired 5FU+CDDP resistant organoids (a) and parental organoids treated with control (CTRL) or 1000U/mL universal type I interferon (IFN) (b). (a) n=3 independent experiments; (b) n=4 independent experiments. Significance were calculated with unpaired two-tailed student t-test. Data was presented as mean ± standard deviation. Source data are provided as a Source Data file.

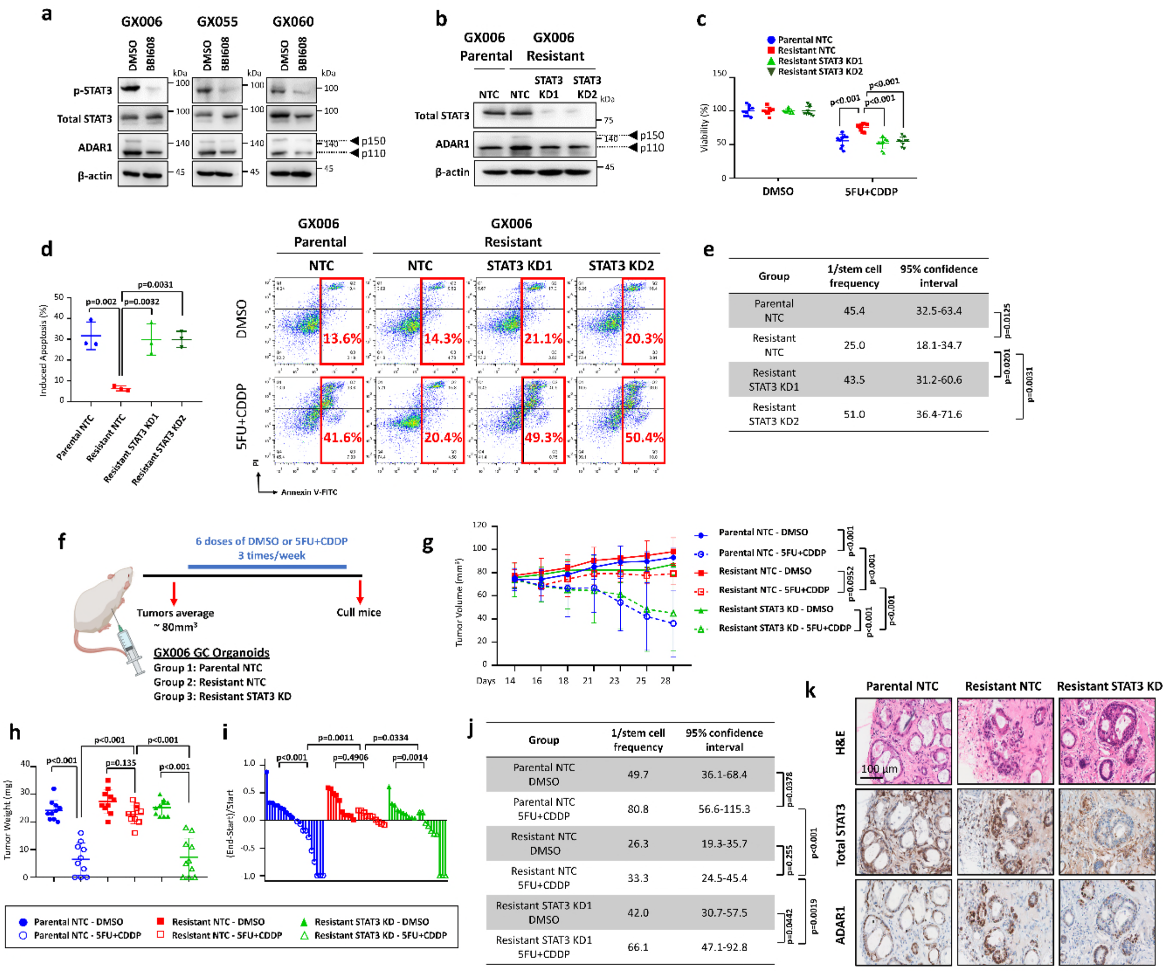

Supplementary Figure 5. a Western blot for phosphorylated and total STAT3, and ADAR1 expression in the three 5FU+CDDP resistant organoid lines in the absence or presence of 2μM STAT3 inhibitor BBI608. b Western blot for total STAT3 and ADAR1 expression in GX006 parental and GX006 5FU+CDDP resistant organoid lines stably transduced with non-target control (NTC) or STAT3 shRNA knockdown (clones 1 and 2). β-actin served as a loading control. c-e CellTiter-Glo analysis (c) and Annexin V-PI analysis (d) showing percentage of apoptotic cells in the absence or presence of 5FU+CDDP, and in vitro limiting dilution spheroid formation and tumor-initiating cell frequency calculation (e) in GX006 parental and GX006 5FU+CDDP resistant organoid lines stably transduced with NTC orSTAT3 shRNA knockdown (clones 1 and 2). f Schematic diagram of treatment regimen comparing GX006 parental organoid lines stably transduced with NTC or STAT3 shRNA knockdown (clone 1) injected subcutaneously into NSG mice. g, h Volume (g) and weight (h) of tumors derived from the indicated cell lines at end point. i Waterfall plot showing the response of each tumor in each group at end point. j Ex vivo limiting dilution assay of HCC tumors harvested from each group to evaluate tumor-initiating cell frequency. k H&E and immunohistochemistry analysis for total STAT3 and ADAR1 in xenografted tumors. Scale bar, 100μm. n=6 randomly captured images were used for analysis. (a-e) n=3 independent experiments. For (f-k), n=10 mice. (k) Significance were

calculated by (c, g) two-way ANOVA; (d, h, i) by one-way ANOVA; (e, j) by one-sided extreme limiting dilution analysis. Data was presented as mean ± standard deviation. NTC for non-target control, STAT3 KD1 and KD2 for shRNA knockdown (clones 1 and 2). ns for not significant. Source data are provided as a Source Data file.

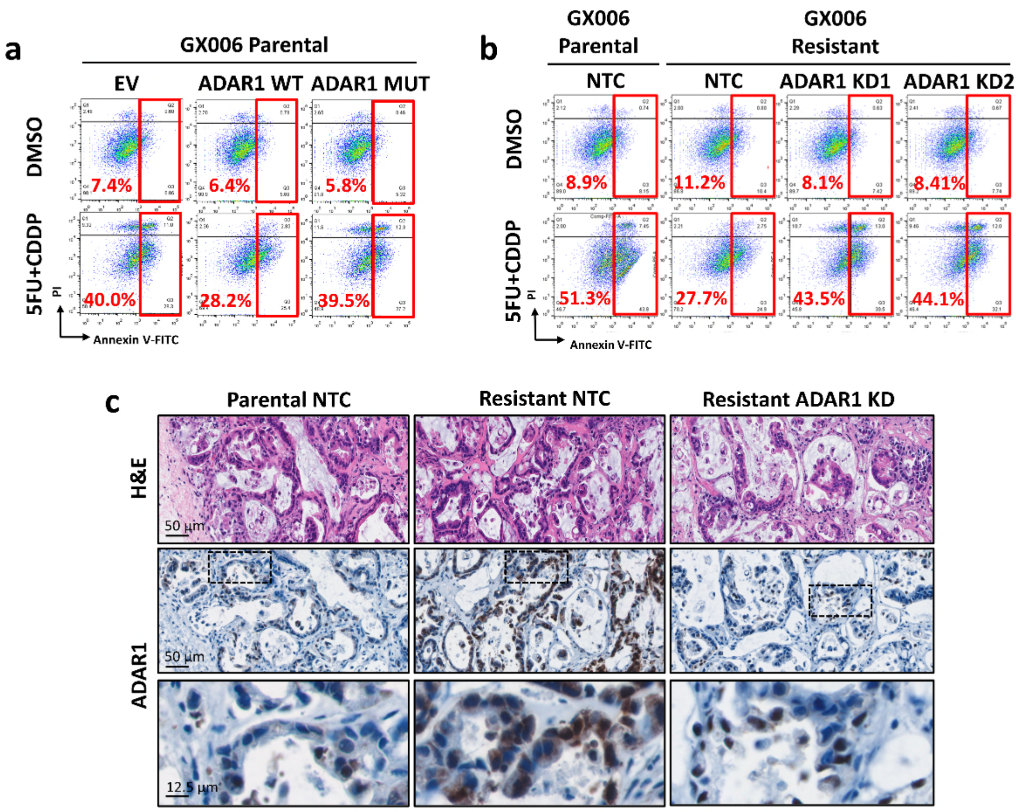

Supplementary Figure 6. a Representative Annexin V-PI apoptosis assays of GX006 parental organoid lines stably transduced with empty vector (EV) control, ADAR1 wild-type (WT) or ADAR1 catalytically-dead mutant (MUT), in the absence or presence of 1.25µM 5FU+5µM CDDP. b Representative Annexin V-PI apoptosis assays of GX006 parental and GX006 5FU+CDDP resistant organoid lines stably transduced with NTC or ADAR1 shRNA knockdown (clones 1 and 2), in the absence or presence of 1.25µM 5FU+5µM CDDP. c H&E and immunohistochemistry analysis for ADAR1 in xenografted tumors. Scale bar, 50µm (low magnification) and 12.5µm (high magnification). n=6 randomly captured images were used for analysis. EV for empty vector control, WT for wild-type, MUT for catalytically-dead mutant, NTC for non-target control, ADAR1 KD1 and KD2 for shRNA knockdown (clones 1 and 2).

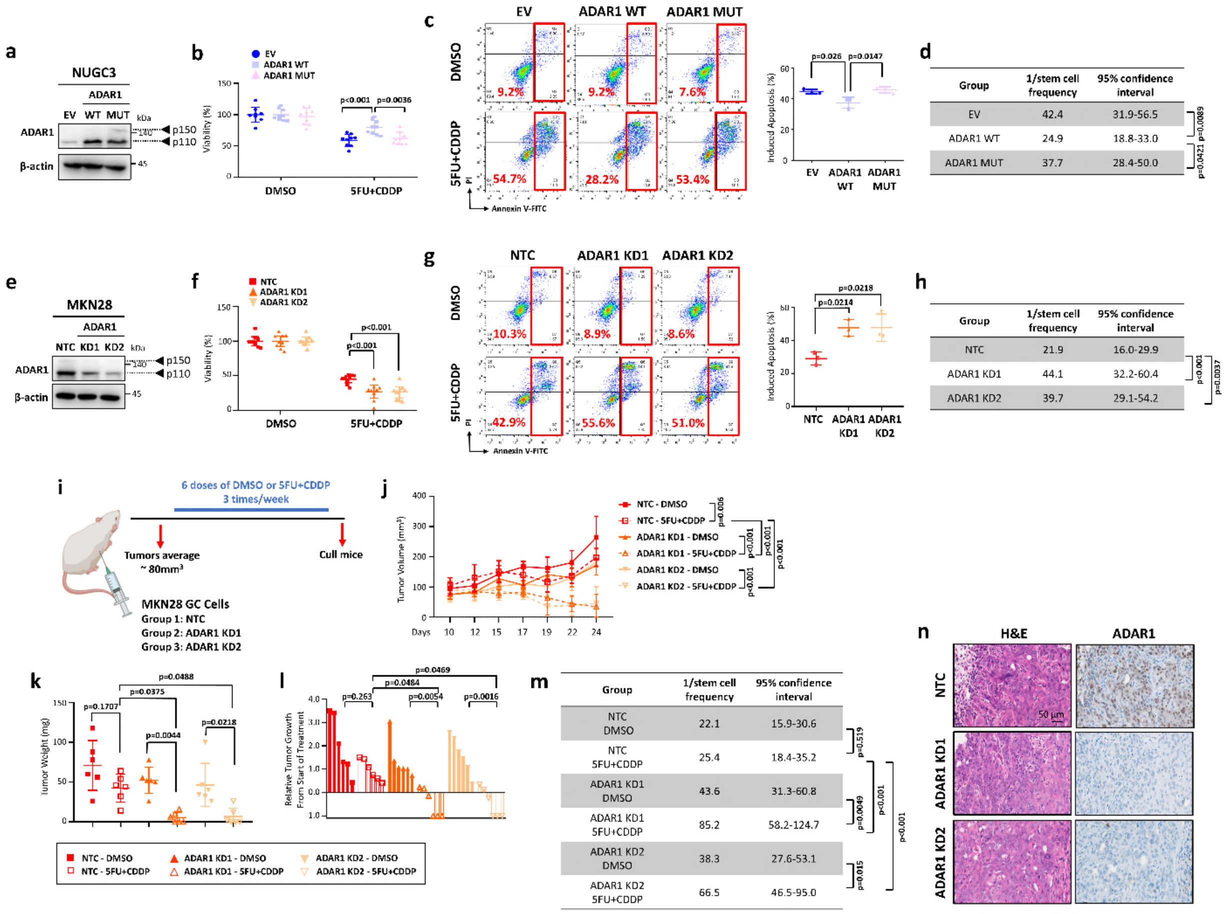

Supplementary Figure 7. a Western blot for ADAR1 in NUGC3 cells stably transduced with empty vector (EV) control, ADAR1 WT or ADAR1 MUT. β-actin served as a loading control. b-d CellTiter-Glo analysis showing viability of cells (b), Annexin V-PI analysis showing percentage of apoptotic cells (c), and in vitro limiting dilution spheroid formation and tumor-initiating cell frequency calculation (d) in NUGC3 cells stably transduced with EV, ADAR1 WT or ADAR1 MUT, in the absence or presence of 10μM 5FU+40μM CDDP. e Western blot for ADAR1 in MKN28 cells stably transduced with non-target control (NTC) or ADAR1 shRNA knockdown (clones 1 and 2). β-actin served as a loading control. f-h CellTiter-Glo analysis showing viability of cells (f), Annexin V-PI analysis showing percentage of apoptotic cells (g), and in vitro limiting dilution spheroid formation and tumor-initiating cell frequency calculation (h) in MKN28 cells stably transduced with NTC or ADAR1 shRNA knockdown (clones 1 and 2), in the absence or presence of 10μM 5FU+40μM CDDP. i Schematic diagram of treatment regimen comparing MKN28 cells stably transduced with NTC or ADAR1 shRNA knockdown (clones 1 and 2) injected into nude mice subcutaneously. j, k Volume (j) and weight (k) of tumors derived from the indicated cell lines at end point. Statistical test for end point noted in the figure. l Waterfall plot showing the response of each tumor in each group at end point. m Ex vivo limiting dilution assay of tumors harvested from each group to evaluate tumor-initiating cell

frequency. n H&E and immunohistochemical analysis for ADAR1 in xenografted tumors. Scale bar, 50µm. n=6 randomly captured images were used for analysis. (a-h) n=3 independent experiments; (i-n), n=6 mice. Significance were calculated by (b, f, j) two-way ANOVA; (c, g, k, l) one-way ANOVA; (d, h, m) one-sided extreme limiting dilution analysis. Data was presented as mean ± standard deviation. EV for empty vector control, WT for wild-type, MUT for catalytically-dead mutant, NTC for non-target control, ADAR1 KD1 and KD2 for shRNA knockdown (clones 1 and 2). ns for not significant. Source data are provided as a Source Data file.

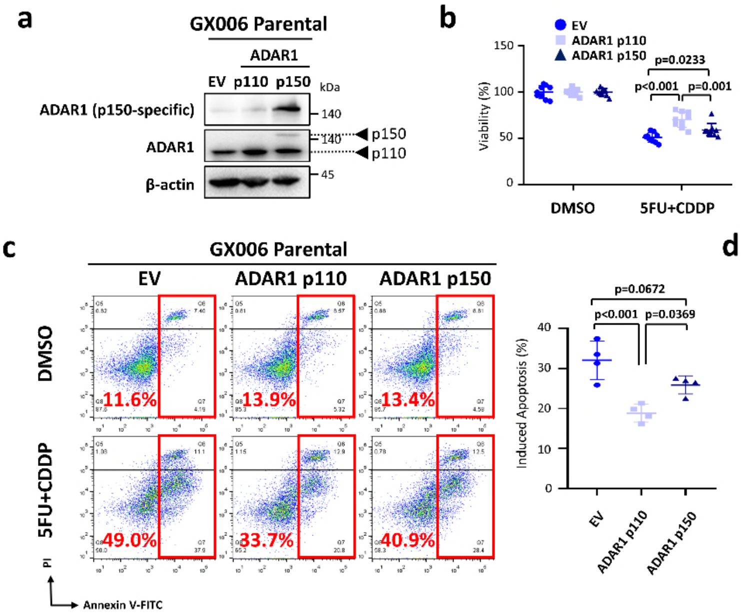

d

Group	1/stem cell frequency	95% confidence interval
Parental EV	38.5	29.1-50.8
Parental ADAR1 p110	22.3	16.9-29.5
Parental ADAR1 p150	33.8	25.6-44.6

p=0.0068 (Parental EV vs Parental ADAR1 p110); p=0.0379 (Parental ADAR1 p110 vs Parental ADAR1 p150); p=0.513 (Parental EV vs Parental ADAR1 p150)

Supplementary Figure 8. a Western blot for ADAR1 p150 and p110 isoform expression in GX006 parental organoid lines stably transduced with empty vector (EV) control, ADAR1 p110 or ADAR1 p150. β-actin served as a loading control. b-d CellTiter-Glo analysis showing viability of cells in the absence or presence of 1.25μM 5FU+5μM CDDP (b), Annexin V-PI analysis showing percentage of apoptotic cells in the absence or presence of 1.25μM 5FU+5μM CDDP (c), and in vitro limiting dilution spheroid formation and tumor-initiating cell frequency calculation (d). For all panels, experiments were performed n=3 independent experiments. Significance were calculated by (b) two-way ANOVA; (c) one-way ANOVA; (d) one-sided extreme limiting dilution analysis. Data was presented as mean ± standard deviation. ns for not significant. Source data are provided as a Source Data file.

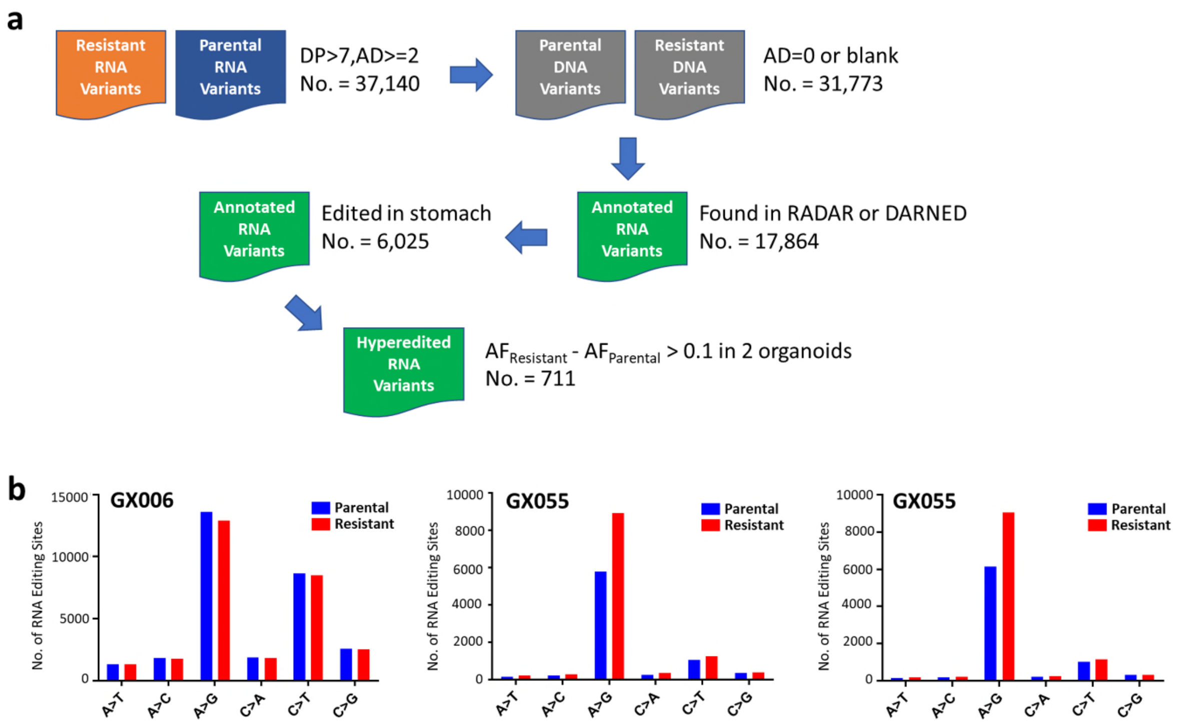

Supplementary Figure 9. a Filtering steps for identification of hyperedited RNA sites in resistant gastric organoids. DP for read depth, AD for allele depth, AF for allele fraction. b Summary of all the RNA variants observed in parental and 5FU+CDDP resistant gastric organoids GX006, GX055 and GX060. All variants observed in DNA were removed based on whole exome sequencing result. Source data are provided as a Source Data file.

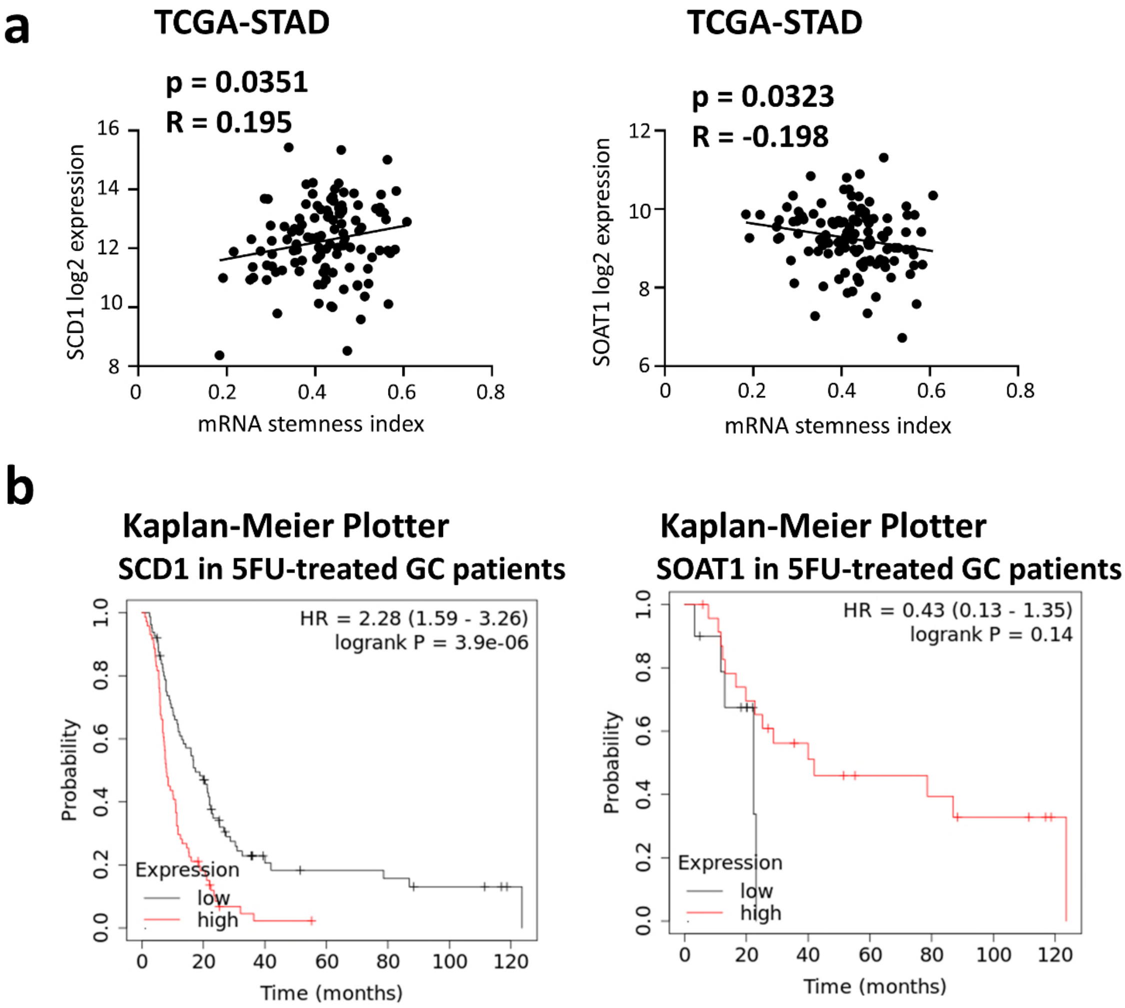

Supplementary Figure 10. a Pearson correlation analysis of mRNA expression of SCD1 (left) or SOAT1 (right), and mRNA stemness index in TCGA-STAD cohort. b Kaplan-Meier overall survival plot comparing 5FU-treated GC patients with high versus low SCD1 expression or high versus low SOAT1 expression using Kaplan-Meier Plotter Gastric Cancer dataset. Sources for the datasets includes GEO, EGA and TCGA. Significance were calculated by (a) two-tailed Pearson correlation analysis; (b) Log-rank test. Source data are provided as a Source Data file.

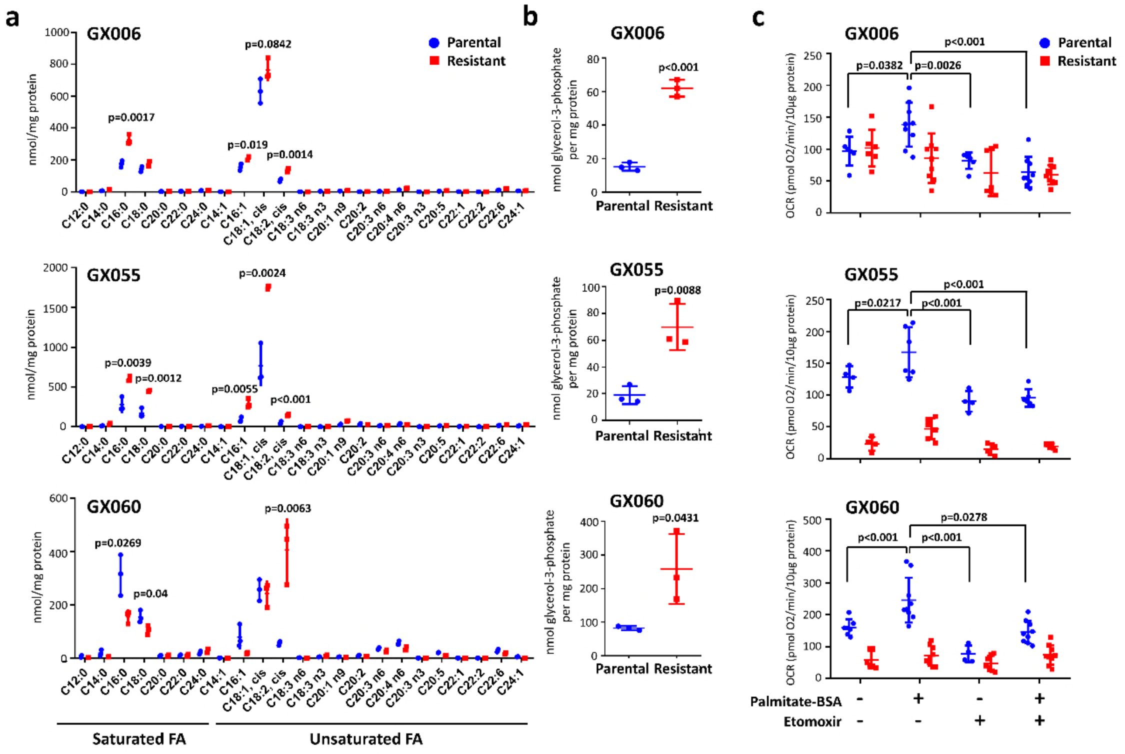

Supplementary Figure 11. a Lipidomic profiling by GC-MS/MS of parental and 5FU+CDDP resistant gastric organoids GX006, GX055 and GX060. n=3 independent biological replicates. Unpaired two-tailed student t-test. b Concentration of glycerol-3-phosphate by GC-MS/MS in parental and 5FU+CDDP resistant gastric organoids GX006, GX055 and GX060. n=3 independent biological replicates. Unpaired two-tailed student t-test. c Rate of lipid oxidation as determined by Seahorse bioenergetic analysis. FA for fatty acid. Experiments were performed minimum of n=4 independent experiments. Significance were calculated by (a, b) unpaired two-tailed student t-test; (c) by one-way ANOVA. Data was presented as mean ± standard deviation. Source data are provided as a Source Data file.

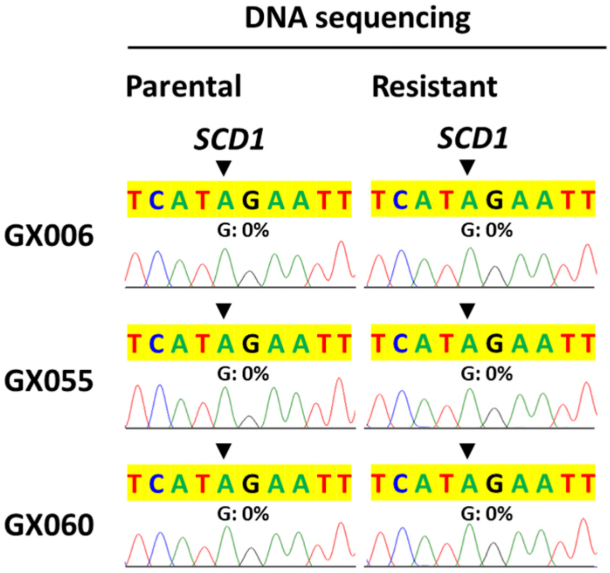

Supplementary Figure 12. Sequence chromatograms of the SCD1 DNA in GX006, GX055 and GX060 GC organoid lines.

Supplementary Figure 13

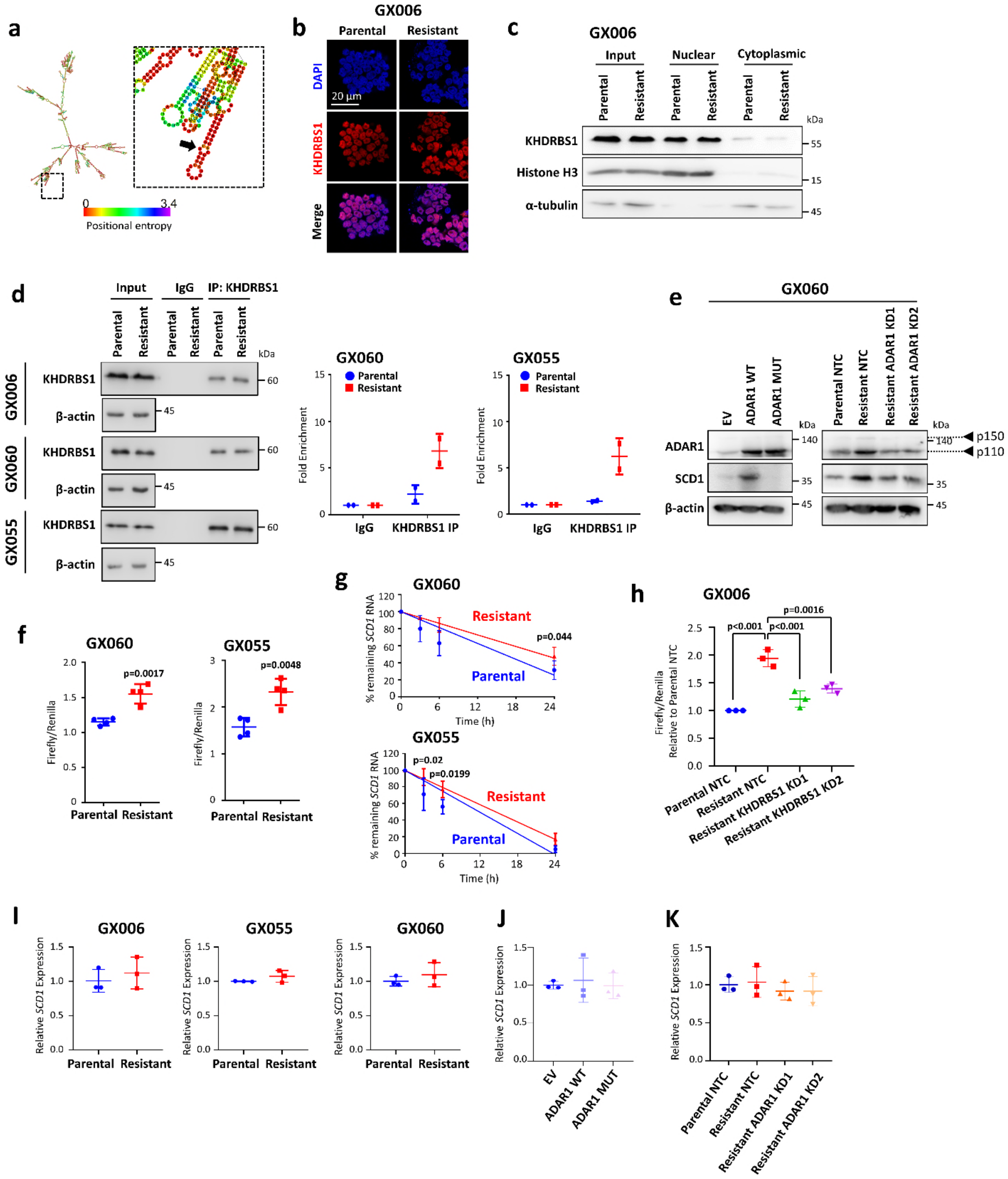

Supplementary Figure 13. a Secondary structure of SCD1 3'UTR predicted by RNAfold. b Immunofluorescence staining of KHDRBS1 in GX006 parental and paired 5FU+CDDP resistant organoids. c Western blot for KHDRBS1 expression following subcellular fractionation of organoids. Histone H3 and α-tubulin were used as control for nuclear and cytoplasmic fractionation, respectively. d RNA immunoprecipitation binding assay of KHDRBS1 in parental or resistant organoids (GX060 and GX055). e Western blot for ADAR1 and SCD1 in parental GX060 organoids

with or without ADAR1 wild-type (WT) and catalytically-dead mutant (MUT) overexpressed; and resistant GX060 organoids with or without ADAR1 repressed. f Luciferase reporter assay with 3'UTR in parental or resistant organoids (GX060 and GX055). g Stability of SCD1 RNA following actinomycin D treatment (10μg/mL) for 3, 6, or 24h as measured by qPCR. h Luciferase reporter assay with SCD1 3'UTR in GX006 parental and GX006 5FU+CDDP resistant organoid lines stably transfected with NTC or KHDRBS1 shRNA knockdown (clones 1 and 2). i-k qPCR analysis of SCD1 expression in parental and paired resistant organoids (i), organoids with ADAR1 overexpressed (j), and organoids with ADAR1 repressed (k). (b-d) n=2 independent experiments; (e, h, i, j, k), n=3 independent experiments; (f) n=4 independent experiments; (g) n=4 (GX055) and n=5 (GX060) independent experiments; Significance were calculated by (f, i) unpaired two-sided student t-test; (g) two-way ANOVA; (h, j, k) one-way ANOVA. Data was presented as mean ± standard deviation. EV for empty vector control, WT for wild-type, MUT for catalytically-dead mutant, NTC for non-target control, ADAR1 KD1 and KD2 for shRNA knockdown (clones 1 and 2). Source data are provided as a Source Data file.

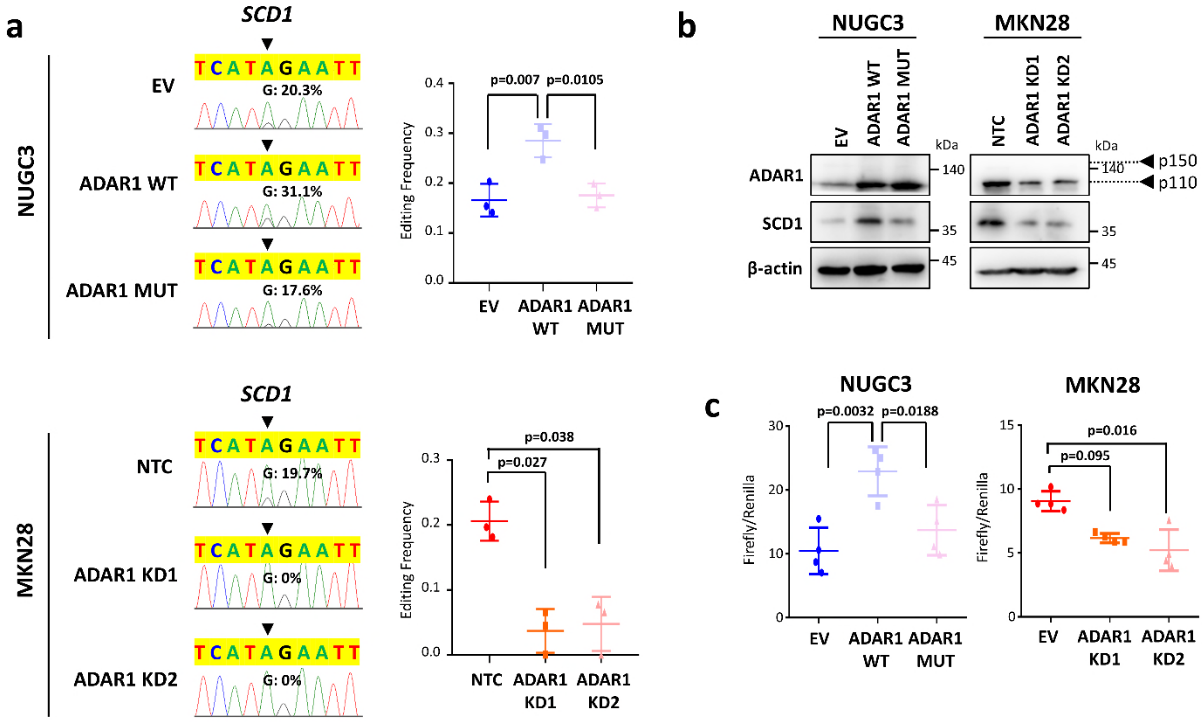

Supplementary Figure 14. a Sequence chromatograms of the SCD1 transcript in the indicated cell groups. Dot plots represent editing levels of SCD1. b Western blot for ADAR1 and SCD1 in NUGC3 cells with or without ADAR1 wild-type (WT) and catalytically-dead mutant (MUT) overexpressed or MKN28 cells with or without ADAR1 repressed. β-actin served as a loading control. Images representative of n=3 independent experiments. c Luciferase reporter assay with 3'UTR in NUGC3 cells with or without ADAR1 WT or MUT overexpressed or MKN28 cells with or without ADAR1 repressed. (a, b) n=3 independent experiments; (c), n=4 independent experiments. Significance were calculated by (a, c) one-way ANOVA. Data was presented as mean ± standard deviation. EV for empty vector control, WT for wild-type, MUT for catalytically-dead mutant, NTC for non-target control, ADAR1 KD1 and KD2 for shRNA knockdown (clones 1 and 2). Source data are provided as a Source Data file.

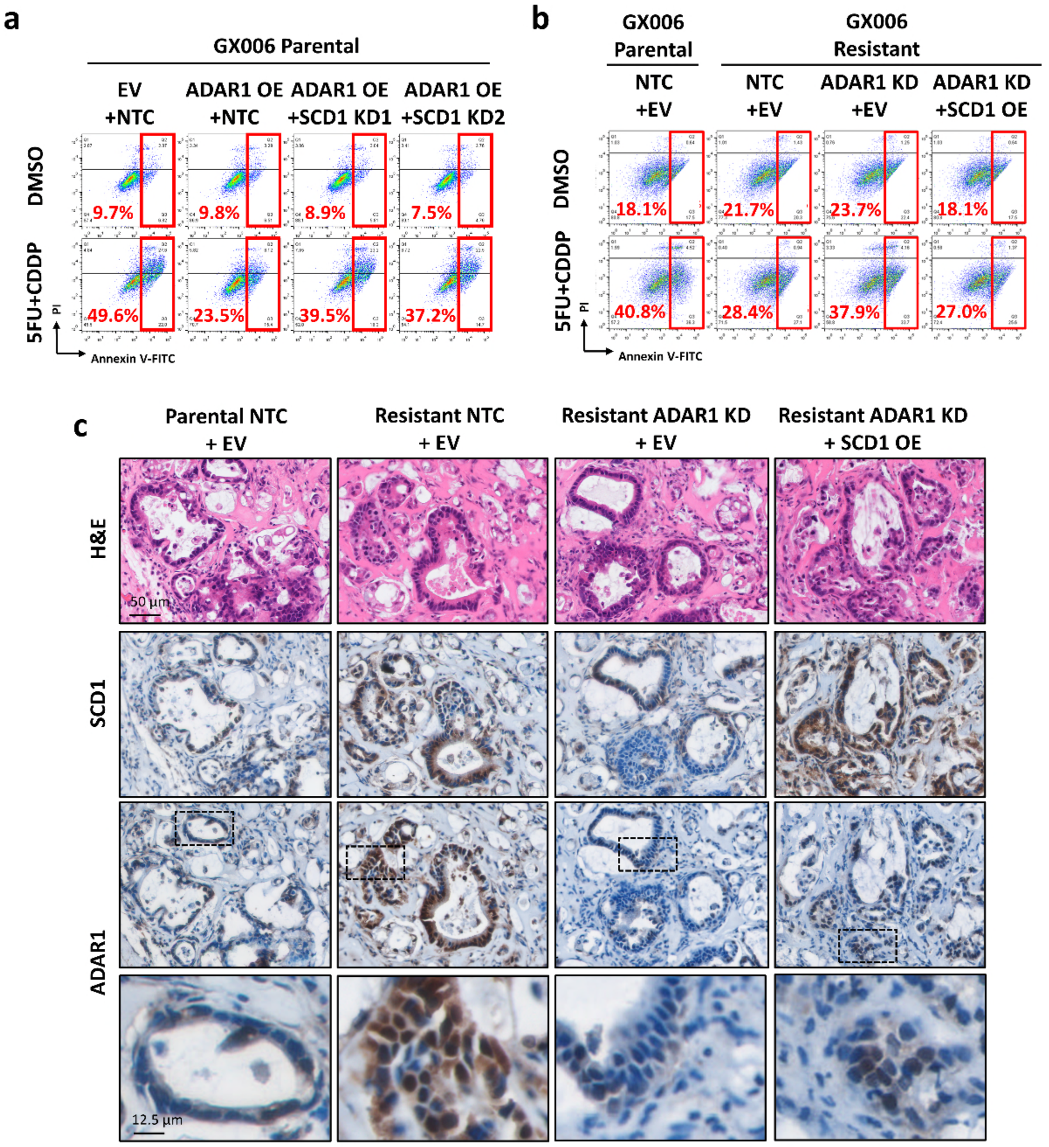

Supplementary Figure 15. a, b Representative Annexin V-PI apoptosis assays of GX006 parental organoid lines with or without ADAR1 overexpressed and with or without SCD1 concomitantly repressed (a) or GX006 parental and GX006 5FU+CDDP resistant organoid lines with or without ADAR1 repressed and with or without SCD1 concomitantly overexpressed (b), in the absence or presence of 1.25μM 5FU+5μM CDDP. c H&E and immunohistochemistry analysis for SCD1 and ADAR1 in xenografted tumors. n=6 randomly captured images were used for analysis. Scale bar, 50μm (low magnification) and 12.5μm (high magnification). EV for empty vector control, OE for overexpression, NTC for non-target control, SCD1 KD1 and KD2 for shRNA knockdown (clones 1 and 2), ADAR1 KD for shRNA knockdown (clone 1).

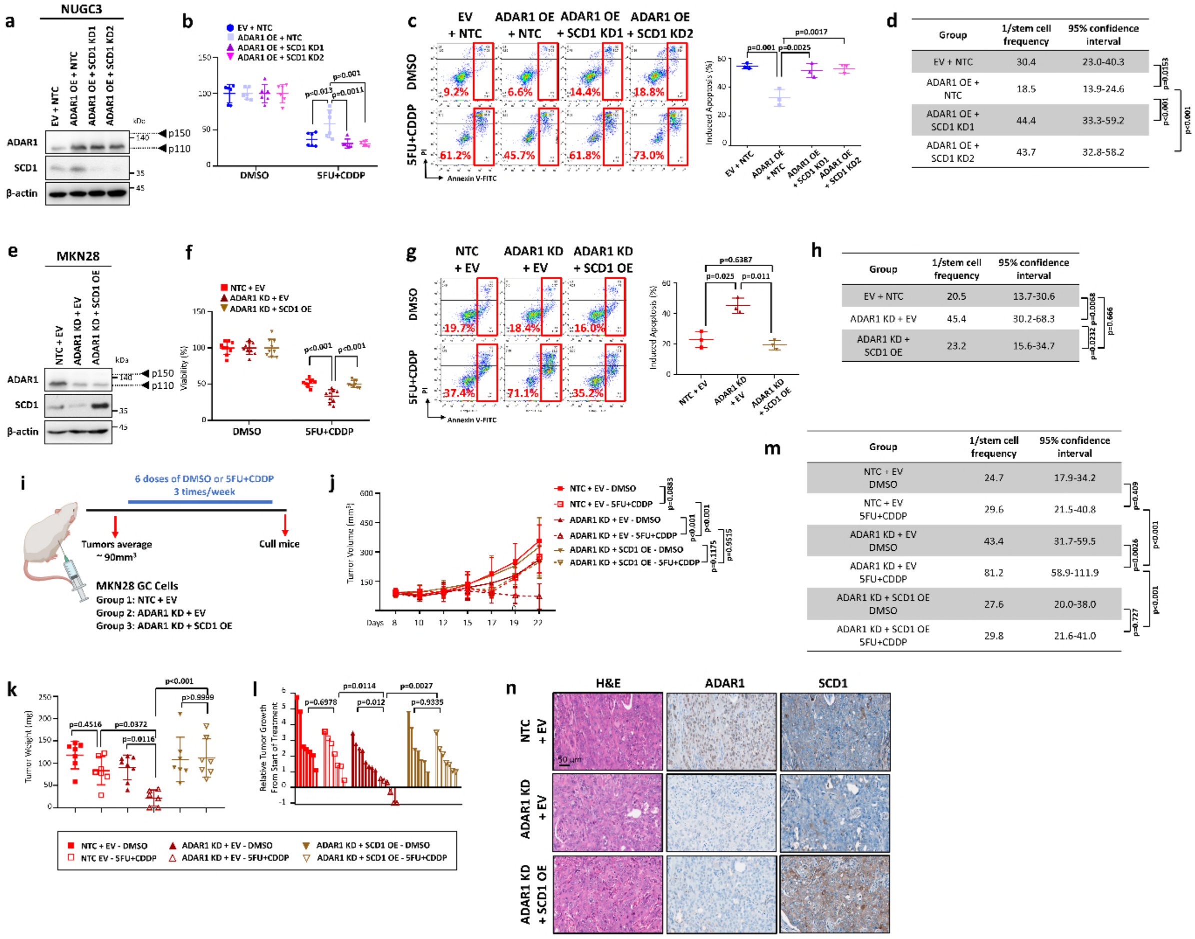

Supplementary Figure 16. a Western blot for ADAR1 and SCD1 expression in NUGC3 cells with or without ADAR1 overexpressed and with or without SCD1 concomitantly repressed. b-d CellTiter-Glo analysis showing viability of cells (b), Annexin V-PI analysis showing percentage of apoptotic cells (c), and in vitro limiting dilution spheroid formation and tumor-initiating cell frequency calculation (d) in NUGC3 cells with or without ADAR1 overexpressed and with or without SCD1 concomitantly repressed e Western blot for ADAR1 and SCD1 expression in MKN28 cells with or without ADAR1 repressed and with or without SCD1 concomitantly overexpressed. f-h CellTiter-Glo analysis showing viability of cells (f), Annexin V-PI analysis showing percentage of apoptotic cells (g), and in vitro limiting dilution spheroid formation and tumor-initiating cell frequency calculation (h) in MKN28 cells with or without ADAR1 repressed and with or without SCD1 concomitantly overexpressed. i Schematic diagram of treatment regimen comparing MKN28 cells with or without ADAR1 repressed and with or without SCD1 concomitantly overexpressed injected into nude mice subcutaneously. j, k Volume (j) and weight (k) of tumors derived from the indicated cell lines at end point. l Waterfall plot showing the response of each tumor in each group at end point. m Ex vivo limiting dilution assay of tumors harvested from each group to evaluate tumor-initiating cell frequency. n H&E and immunohistochemical analysis for ADAR1 and SCD1 in xenografted tumors.

Scale bar, 50µm. (a-h) n=3 independent experiments; (i-n), n=6-8 mice. Significance were calculated by (b, f, j) two-way ANOVA; (c, g, k, l) one-way ANOVA; (d, h, m) one-sided extreme limiting dilution analysis. Data was presented as mean ± standard deviation. EV for empty vector control, NTC for non-target control, OE for overexpression, SCD1 KD1 and KD2 for SCD1 shRNA knockdown (clones 1 and 2), ADAR1 KD for ADAR1 shRNA knockdown (clone 1). ns for not significant. Source data are provided as a Source Data file.

a

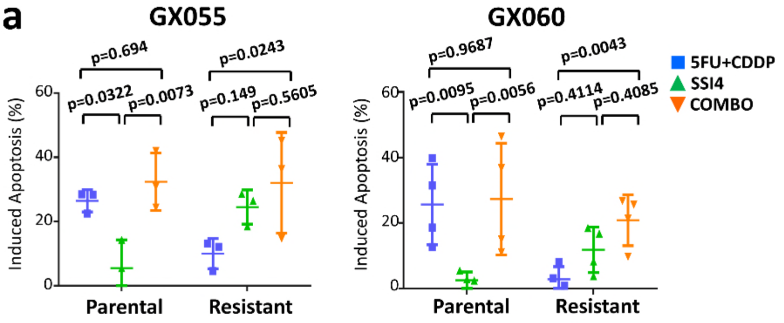

b

GX055

Group	1/stem cell frequency	95% confidence interval	
Parental + DMSO	60.3	44.5-81.6	p=0.817
Parental + SSI4	63.4	46.7-86.2	
Resistant + DMSO	37.5	28.2-49.8	p<0.001
Resistant + SSI4	96.3	68.4-135.7	

GX060

Group	1/stem cell frequency	95% confidence interval	
Parental + DMSO	48.0	34.3-67.1	p=0.323
Parental + SSI4	62.1	43.7-88.2	
Resistant + DMSO	28.4	20.6-39.4	p=0.0077
Resistant + SSI4	55.8	39.5-78.7	

Supplementary Figure 17. a, b Functional assays investigating the effect of pharmacological inhibition of SCD1 using a SCD1 specific inhibitor SSI4 in GX055 and GX060 parental and 5FU+CDDP resistant organoid lines. Comparisons include DMSO versus 5FU+CDDP versus SSI4 versus combination of 5FU+CDDP and SSI4 (COMBO) using Annexin V-PI apoptosis (a) and in vitro limiting dilution spheroid formation assays (b). (a) n=3 (GX055), n=4 (GX060) independent experiments; (b) n=3 independent experiments. Significance were calculated by (a) two-way ANOVA; (b) one-sided extreme limiting dilution analysis. Data was presented as mean ± standard deviation. COMBO for combination. ns for not significant. Source data are provided as a Source Data file.

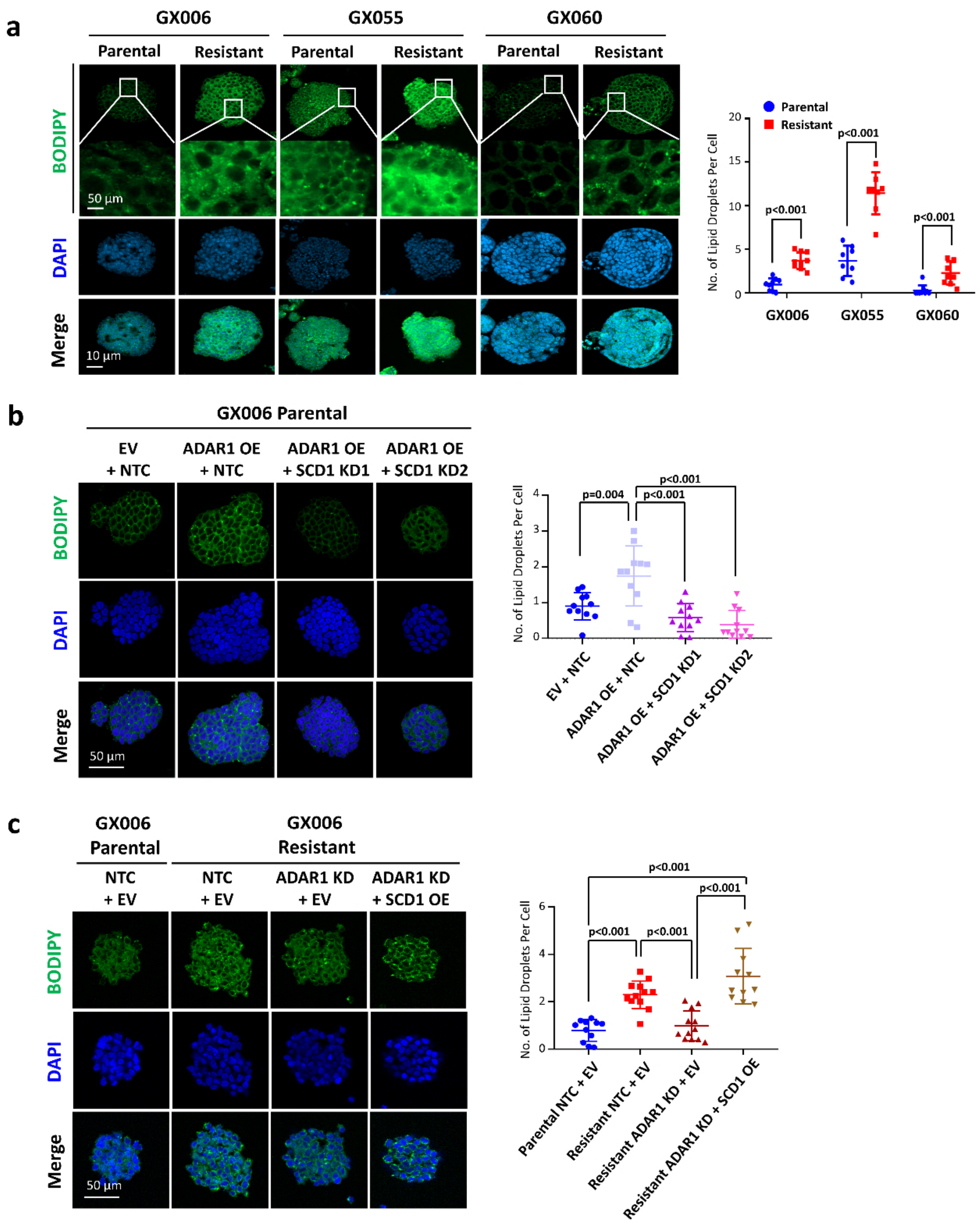

Supplementary Figure 18. a Representative immunofluorescence images and quantification of BODIPY staining of lipid droplet in parental and resistant GX006, GX055 and GX060 GC organoids. Scale bar, 50μm. Scale bar in magnified image, 10μm. b Representative immunofluorescence images and quantification of ER tracker staining in GX006 organoids with or without ADAR1 overexpressed and with or without SCD1 concomitantly repressed. Scale bar, 50μm. c Representative immunofluorescence images and quantification of BODIPY staining of lipid droplet in GX006 organoids with or without ADAR1 overexpressed and with or without SCD1 concomitantly repressed. Scale bar, 50μm. EV for empty vector control, NTC for non-target control, OE for

overexpression, SCD1 KD1 and KD2 for SCD1 shRNA knockdown (clones 1 and 2), ADAR1 KD for ADAR1 shRNA knockdown (clone 1). (a) n=3 independent experiments; (b) n=3 independent experiments; (c) n=3 independent experiments. Significance were calculated by (a) unpaired two-tailed student t-test; (b, c) one-way ANOVA. Data was presented as mean ± standard deviation. Source data are provided as a Source Data file.

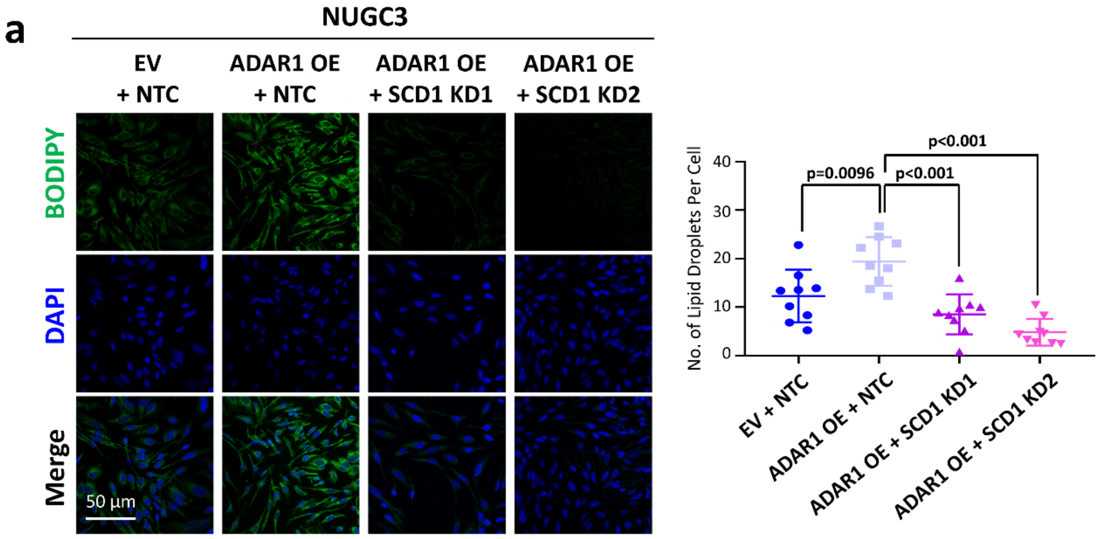

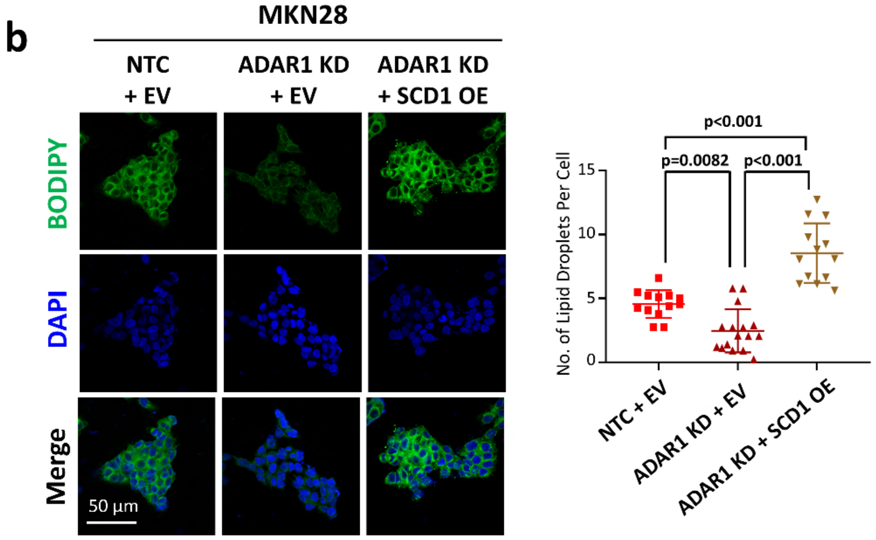

Supplementary Figure 19. a, b Representative immunofluorescence images and quantitation of BODIPY staining to estimate lipid droplet numbers in NUGC3 cells with ADAR1 overexpression and SCD1 concomitantly repressed (a), and MKN28 cells with ADAR1 repressed and SCD1 concomitantly overexpressed (b). DAPI to mark the nucleus (a) n=3 independent experiments; (b) n=3 independent experiments. Significance were calculated by one-way ANOVA. Data was presented as mean ± standard deviation. EV for empty vector control, NTC for non-target control, OE for overexpression, SCD1 KD1 and KD2 for SCD1 shRNA knockdown (clones 1 and 2), ADAR1 KD for ADAR1 shRNA knockdown (clone 1). Scale bar, 50µmSource data are provided as a Source Data file.

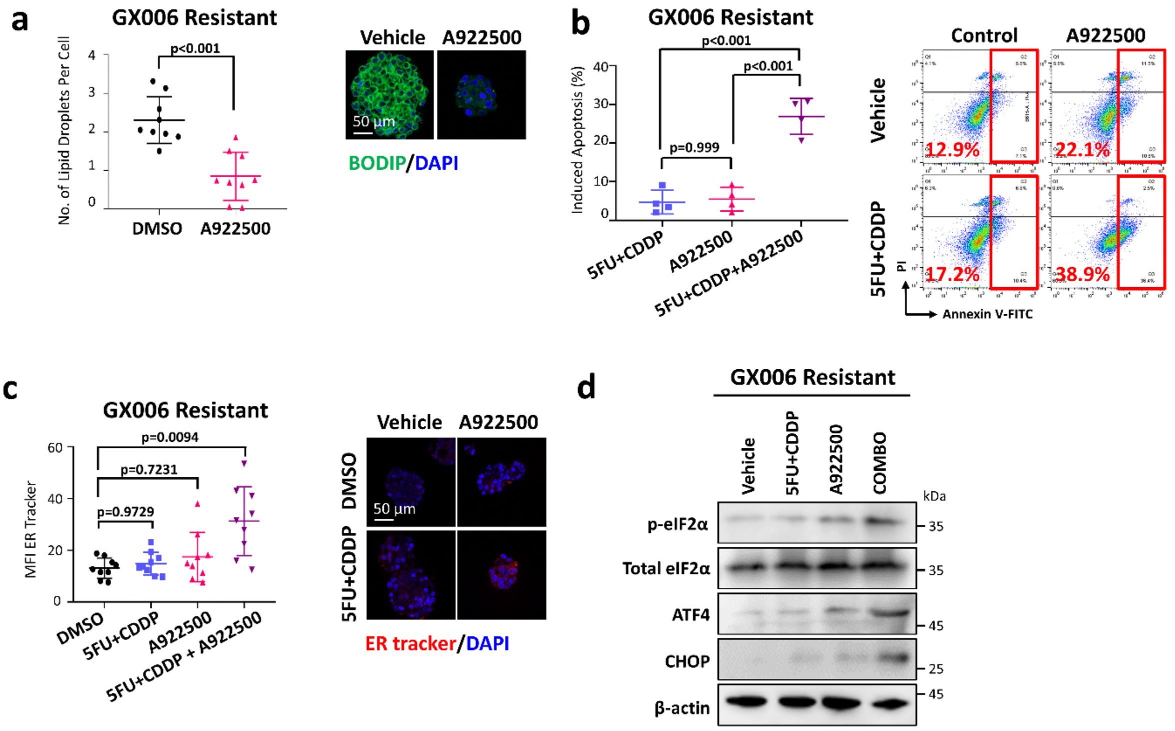

Supplementary Figure 20. a Representative immunofluorescence images and quantitation of BODIPY staining to estimate lipid droplet numbers in GX006 resistant organoid treated with vehicle control (DMSO) or lipid droplet formation inhibitor 10µM A922500 for 48h. Scale bar, 50µm. b Annexin V-PI analysis showing percentage of apoptotic cells treated with DMSO or 1.25µM 5FU+5µM CDDP and in combination with vehicle or 10µM A922500. c Representative immunofluorescence images and quantification of ER tracker staining in the indicated treatment groups in GX006 5FU+CDDP resistant organoids. One-way ANOVA. Scale bar, 50µm. d Western blot analysis for expression of ER stress markers including total and phosphorylated eIF2α, ATF4 and CHOP upon indicated treatment groups in GX006 5FU+CDDP resistant organoids. β-actin is used as loading control. (a) n=3 independent experiments; (b) n=4 independent experiments; (c) n=3 independent experiments; (d) n=3 independent experiments. Significance were calculated by (a) unpaired two-tailed student t-test; (b, c) one-way ANOVA. Data was presented as mean ± standard deviation. ns for not significant. Source data are provided as a Source Data file.

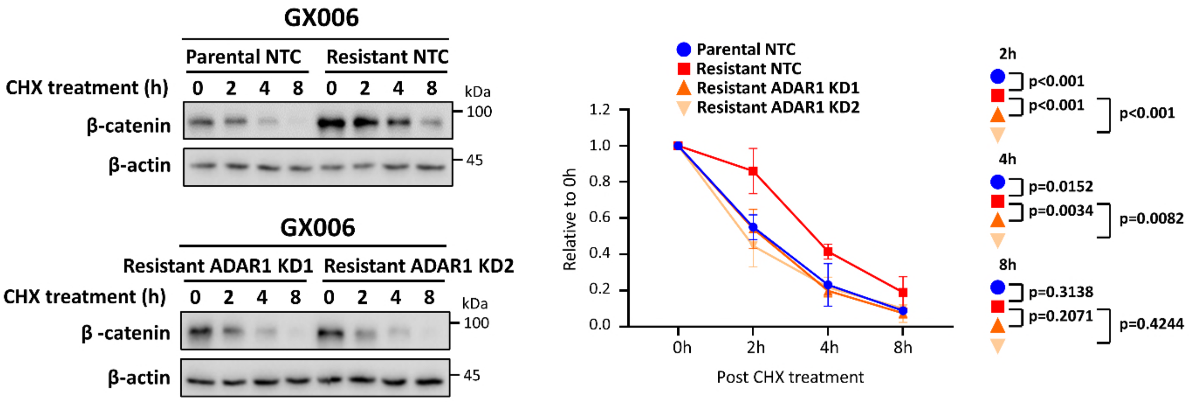

Supplementary Figure 21. Western blot for β-catenin following cycloheximide (CHX) treatment for 0,2,4, and 8h in GX006 parental and GX006 5FU+CDDP resistant organoids stably transduced with non-target control (NTC) or ADAR1 shRNA knockdown (clones 1 and 2). β-actin served as a loading control. Images representative of n=3 independent experiments. Significance were calculated by two-way ANOVA. NTC for non-target control, ADAR1 KD1 and KD2 for shRNA knockdown ADAR1 (clones 1 and 2). Data was presented as mean ± standard deviation. Source data are provided as a Source Data file.

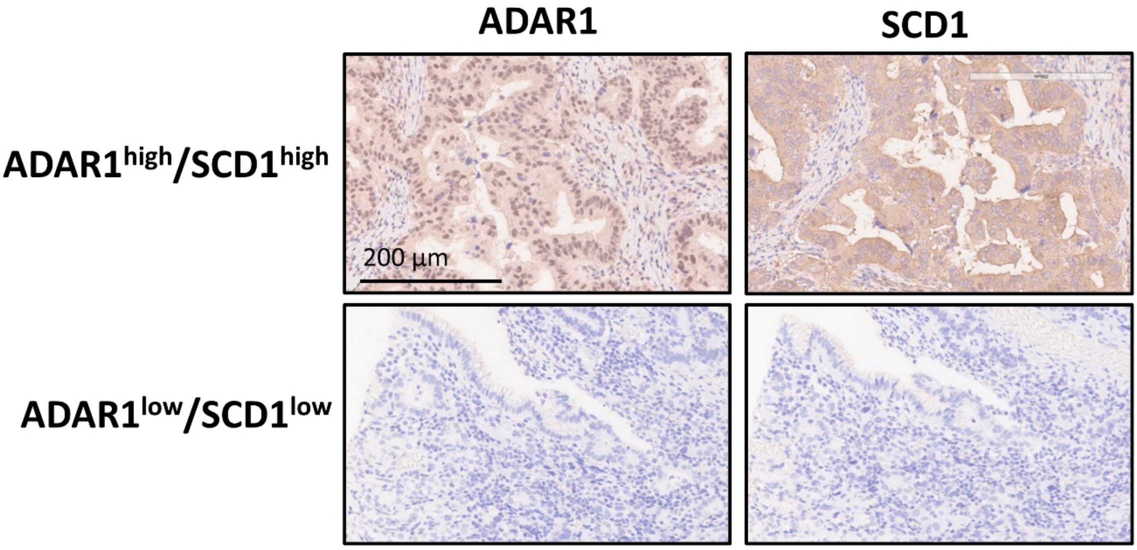

Supplementary Figure 22. Representative immunohistochemical staining of ADAR1 and SCD1 in gastric cancer patients with high ADAR1 and high SCD1 expression versus low ADAR1 and low SCD1 expression. $ADAR1^{low}$; $SCD1^{low}$ (n=23), $ADAR1^{high}$; $SCD1^{high}$ (n=19). Scale bar, 200µm.

Supplementary Table 1. List of bioinformatic analysis tools used in this study.

Tools
ANNOVAR[1]
BWA[2]
FACETS[3]
GATK UnifiedGenotyper[4,5,6]
GATK HaplotypeCaller[4,5,6]
RSEM[7]
Samtools[8]
SomaticIndelDetector[4,5,6]
STAR[9]
Strelka[10]

Supplementary Table 2. PCR primers used in this study.

Gene	Primer
Human AXIN2	F (5'- CAGCGAGTATTACTGCTACTCGAAA -3')
	R (5'- TTTTTTGTGCTTTGGGCACTATG -3')
Human CCND1	F (5'- GATGCCACCCTCCTCAACGA -3')
	R (5'- GGAAGCGGTCCAGGTAGTTC -3')
Human LRP5	F (5'- GGGAGACGCCAAGACAGACAAGATCG -3')
	R (5'- GGTGAAGACCAAGAAGGCCTCAGG -3')
Human LRP6	F (5'- CCCATGCACCTGGTTCTACT -3')
	R (5'- CTGGAACTGGGACTCTGAGC -3')
Human CTGF	F (5'- CTTGCGAAGCTGACCTCGAAGA -3')
	R (5'- CCGTCGGTACATACTCCACAG -3')
Human CYR61	F (5'- CGCCTTGTGAAAGAAACCCG -3')
	R (5'- GGTTCGGGGGATTTCTTGGT -3')
Human ALDH1A1	F (5'- CGGCGCATTGTGTTAGCTGATGCCG -3')
	R (5'- AGAGAACACTGTGGGCTGGAC -3')
Human NANOG	F (5'- AATACCTCAGCCTCCAGCAGATG -3')
	R (5'- TGCGTCACACCATTGCTATTCTTC -3')
Human *β*-ACTIN	F (5'- CATCCACGAAACTACCTTCAACTCC -3')
	R (5'- GAGCCGCCGATCCACACG -3')
Human SCD1	F (5'- CTCCTGGCTCGGGGTAAAAA -3')
	R (5'- TAGAGGGGCATCGTCTCCAA -3')
Human ADAR1	F (5'-TGCTGCTGAATTCAAGTTGG -3')
	R (5'-TCGTTCTCCCCAATCAAGAC -3')
Human p110 ADAR1	F (5'- GACTGAAGGTAGAGAAGGCTACG -3')
	R (5'- TGCACTTCCTCGGGACAC -3')
Human p150 ADAR1	F (5'- CGGGCAATGCCTCGC -3')
	R (5'- AATGGATGGGTGTAGTATCCGC-3')
SCD1 A-to-I editing	F (5'- CTCCTGGCTCGGGGTAAAAA -3')
	R (5'- TTTGACCCGACTTCACCTCC -3')

Supplementary Data 1. List of hyperedited A-to-I editing sites found in 5FU+CDDP resistant GC organoids.

Please refer to additional supplementary data file.

Supplementary References

1. Wang K., Li M., Hakonarson H. ANNOVAR: functional annotation of genetic variants from high-throughput sequencing data. Nucleic Acids Res. 38, e164 (2010).

2. Li H., Durbin R. Fast and accurate long-read alignment with Burrows-Wheeler transform. Bioinformatics 26, 589-595 (2010).

3 Shen R., Seshan V. E. FACETS: allele-specific copy number and clonal heterogeneity analysis tool for high-throughput DNA sequencing. Nucleic Acids Res. 44, e131 (2016).

4. McKenna A. et al. The Genome Analysis Toolkit: a MapReduce framework for analysing next-generation DNA sequencing data. Genome Res. 20, 1297-1303 (2010).

5. DePristo M. A. et al. A framework for variation discovery and genotyping using next-generation DNA sequencing data. Nat Genet. 43, 491-498 (2011).

6. Van der Auwera G. A. et al. From FastQ data to high confidence variant calls: the Genome Analysis Toolkit best practices pipeline. Curr. Protoc. Bioinformatics 43,11.10.1-11.10.33 (2013).

7. Li B., Dewey C. N. RSEM: accurate transcript quantification from RNA-seq data with or without a reference genome. BMC Bioinformatics 12, 323 (2011).

8. Heng L. et al. The sequence alignment/map format and SAMtools. Bioinformatics 25, 2078-2079 (2009).

9. Dobin A. et al. STAR: ultrafast universal RNA-seq aligner. Bioinformatics 29, 15-21 (2013).

10. Saunders C. T. et al. Strelka: accurate somatic small-variant calling from sequenced tumor-normal sample pairs. Bioinformatics 28, 1811-1817 (2012).
