# Supplementary material for: ADAR1-mediated RNA editing of SCD1 drives drug resistance and self-renewal in gastric cancer
Source: Nat Commun. 2023 May 19;14:2861. doi: 10.1038/s41467-023-38581-8 (PMC10199093; doi:10.1038/s41467-023-38581-8)
Supplement: Supplementary file 3 — Description of Additional Supplementary Files [file 41467_2023_38581_MOESM3_ESM.pdf]

Description of Additional Supplementary Files

File Name: Supplementary Data 1
Description: List of hyperedited A-to-I editing sites found in 5FU+CDDP resistant gastric organoids.
